# Comparative genomics revealed the gene evolution and functional divergence of *TaMRS2/CorA/NIPA* magnesium transporter families in wheat (*Triticum aestivum L.*)

**DOI:** 10.3389/fpls.2026.1737134

**Published:** 2026-03-09

**Authors:** Xiang-Yang Hao, Yu Zhang, Jian-Zhou Zhang, Hai-Bin Dong, Chao-Jun Peng, Xi-Jun Du, Chun-Ping Wang, Yi-Han Fu, Xue-Li Qi

**Affiliations:** 1 College of Agronomy, Henan University of Science and Technology, Luoyang, Henan, China; 2 National Engineering Laboratory of Wheat, Key Laboratory of Wheat Biology and Genetic Breeding in Central Huanghuai Area, Ministry of Agriculture, Henan Key Laboratory of Wheat Germplasm Resources Innovation and Improvement, Institute of Crops Molecular Breeding, Henan Academy of Agricultural Sciences, Zhengzhou, Henan, China; 3 The Shennong Laboratory, Zhengzhou, Henan, China

**Keywords:** comparative analysis, expression analysis, gene duplication events, magnesium, magnesium transporter, wheat

## Abstract

**Introduction:**

Magnesium transporters (MGTs) are crucial for Mg²⁺ uptake, transport, and storage. Although MGT family has been characterized in many plants, genome-wide identification and functional analysis of MGTs in wheat (*Triticum aestivum L*.) remain largely unclear.

**Methods:**

In this study, we identified wheat *MGT* genes using comparative genomics, and further analyzed their phylogeny, gene structure, conserved motifs, subcellular localization, gene duplication, protein–protein interactions, and expression patterns. Tissue-specific expression was verified by qRT‑PCR.

**Results:**

A total of 63 *TaMGT* genes were identified and classified into MRS2, CorA, and NIPA subfamilies. Most TaMGT proteins were predicted to target membrane systems. A total of 200 magnesium transporter genes were found considered to be generated by gene duplication events, and 1045 interacting proteins were predicted. *TaMGT* genes were highly expressed in flowering anthers. qRT‑PCR confirmed that 24 *TaMRS2* genes exhibited obvious tissue specificity, with higher expression in leaves, stems, and spikes than in roots.

**Discussion:**

This study systematically reveals the evolutionary characteristics and functional differentiation of wheat *MGT* genes, and supports their important roles in reproductive growth. Our results provide a theoretical basis for further functional studies of magnesium transporters in wheat.

## Introduction

Magnesium (Mg) is the second most abundant cation in plants and the fourth most abundant element in vertebrates, playing a crucial role in the growth and development of both plants and animals (Maguire and Cowan, 2002; Tian et al., 2021; Kumar et al., 2024). However, Mg present in soil is not readily absorbable by plants due to its crystalline structure. In contrast, magnesium in the form of Mg^2+^ serves as an essential macronutrients for plants, ensuring their normal development and successful reproduction (de Sousa Ferreira et al., 2023; Ahmed et al., 2023). For instance, For instance, Mg²^+^ acts as the central atom in the porphyrin ring of chlorophyll, which is indispensable for plants to carry out photosynthesis. Additionally, Mg²^+^ enhances membrane stability, participates in the regulation of ion transport, and functions as a coenzyme in the Mg²^+^-ATP complex, thereby activating over 300 enzymes involved in basic physiological processes (Hansson et al., 2013; Gout et al., 2014; Borah and Bhuyan, 2017). Furthermore, Mg^2+^ is implicated in nucleic acid synthesis and protein synthesis. Deficiency in Mg^2+^ can induce adverse effects in plants, such as interveinal chlorosis and retarded growth of roots and leaves (Tanoi and Kobayashi, 2015; Huang et al., 2019; Li et al., 2024). Long-term magnesium deficiency leads to excessive starch accumulation and plant chlorosis, which further reduces the photosynthetic rate and impairs plant growth (Guo et al., 2016; Chaudhry et al., 2021). Meanwhile, several studies have demonstrated that Mg^2+^ can also reduce the toxicity of heavy metals such as Al^3+^, Cu^2+^, and Cd^2+^ to plants (Rengel et al., 2015; Moustaka et al., 2016). However, despite the multiple integral functions of Mg^2+^ in plants, there is also evidence that excess Mg^2+^ is also toxic to plants (Tang et al., 2022). Therefore, maintaining magnesium ion homeostasis in plants is crucial for their growth and development.

Magnesium transporters (MGTs) are a class of proteins that play a crucial role in the absorption, transport, and storage of magnesium (Ishijima, 2021). These proteins contain two or more transmembrane (TM) domains in their C-terminal region. Previous bacterial studies identified three different types of Mg^2+^ transporters, cobalt (Co^2+^) resistant A (CorA), Mg^2+^ transporter (Mgt) A/B, and MgtE (Pohland and Schneider, 2019). In yeast, five CorA congeners with distinct functions have been identified, and these five proteins act synergistically to maintain Mg^2+^ homeostasis in yeast cells (Ishijima, 2021; Franken et al., 2022). In plants, CorA homologs were first identified in *Arabidopsis thaliana* and named *AtMHX1* (Conn et al., 2011). Among the identified MRS2/MGT family members in *Arabidopsis thaliana*, the conserved Gly-Met-Asn (GMN) motifs in two *MRS2/MGT* genes (*AtMRS2-8* and *AtMRS2-9*) have mutated into AMN or GIN motifs, rendering these genes pseudogenes (Gebert et al., 2009). To date, members of the *MRS2/MGT* gene family have been identified in several species, including rice, maize, pear, sugarcane, citrus, *brassica rape*, *camelina sativa*, grape and apple (Saito et al., 2013; Li et al., 2016; Zhao et al., 2018; Wang et al., 2019; Liu et al., 2019; Zhang et al., 2019; Faraji et al., 2021; Ge et al., 2022; Bao et al., 2025). Based on their sequence and structure, Mg^2+^ transporter proteins can be classified into three groups: MRS2, CorA, and NIPA (Faraji et al., 2021). MRS2 and CorA proteins contain two transmembrane (TM) domains in their C-terminal regions, with a conserved GMN tripeptide motif located near the end of the first TM domain, while NIPAs contain several TMs in their structures. These TM are essential for Mg^2+^ transport (Knoop et al., 2005; Faraji et al., 2021).

Multiple studies have demonstrated that the loss of function of magnesium transporters impairs plant growth and development. For example, the loss of function of AtMGT10 impairs chloroplast development in *Arabidopsis thaliana*, while the loss of function of AtMGT9 results in the abortion of mature pollen grains, leading to male sterility (Dukic et al., 2023; Chen et al., 2009). Nine members of the *Arabidopsis* MRS2/MGT family have been reported to exhibit Mg^2+^ transport capacity in complementation assays using yeast *mrs2* mutants (Gebert et al., 2009). In recent years, several studies have shown that some members of this family have specific physiological functions, including tissue-specific expression in roots, stems, leaves, and flowers. Most critically, MRS2/MGT family members play a key role in the translocation of magnesium from roots to buds and the transport of magnesium within cellular compartments (Zhang et al., 2019; Faraji et al., 2021; Ge et al., 2022). For instance, the *AtMRS2-6/AtMGT5, AtMRS2-2/AtMGT9*, and *AtMRS2-3/AtMGT4* genes are vital for pollen mitosis and magnesium supply during pollen formation (Li et al., 2015). AtMRS2-11/AtMGT10 is localized to the chloroplast envelope and may be involved in Mg^2+^ translocation into chloroplasts and chlorophyll (Chl) metabolism (Dukic et al., 2023). AtMRS2-1/AtMGT2 and AtMRS2-5/AtMGT3 are thought to participate in the transport of magnesium ions in vacuoles, thereby maintaining Mg^2+^ homeostasis in mesophyll cells (Saito et al., 2013). AtMRS2-10/AtMGT1 is involved in root Mg²^+^ uptake and exhibits high affinity for Mg²^+^; overexpression of *AtMRS2-11/AtMGT10* in tobacco can increase Mg²^+^ accumulation and enhance the competitiveness of Mg²^+^ against aluminum (Al) stress (Liang et al., 2017; Ishijima, 2021). In other crops, *OsMGT1* in rice has been shown to be involved in root Mg²^+^ uptake (Chen et al., 2017). In maize, *ZmMGT10* responds to magnesium deficiency and confers low magnesium tolerance when heterologously expressed in transgenic *Arabidopsis* (Li et al., 2017). In summary, the MRS2/MGT magnesium transporter family is widely distributed and functionally conserved in plants. Different members of this family profoundly influence plant growth and development (e.g., pollen formation and chloroplast development) and stress responses by regulating Mg²^+^ transport and homeostasis in various tissues or organelles.

The NIPA family is also a core group of transporter proteins involved in regulating plant Mg²^+^ homeostasis (Goytain et al., 2008). Unlike CorA/MRS2-MGT transporters, NIPA proteins are mainly localized to the cell membrane of root cells and possess both high-affinity Mg²^+^ uptake activity and long-distance transport functions from roots to stems and leaves. These three transporter families (CorA, MRS2-MGT and NIPA) have conserved functions, with distinct division of labor and synergistic effects, collectively forming a complete regulatory network for Mg²^+^ in plants. Unfortunately, research on the functional validation of NIPA family members in crops is relatively backward at present.

Wheat is the world’s most extensively cultivated, highly productive, and widely distributed food crop. Mg^2+^ deficiency affects many metabolic processes in wheat, such as Chl synthesis, carbon fixation, photosynthesis, and the transport of carbohydrate sources to sinks, ultimately resulting in yield loss (Yilmaz et al., 2017). Although Mg^2+^ transporters is important in Mg^2+^ homeostasis, systematic and comprehensive research on Mg^2+^ transporters in wheat has not been reported to date. This study takes genome-wide analysis as the core approach, aiming to fill the gap in the systematic research on the magnesium transporter family in wheat. Through systematic identification of family members, clarification of their subfamily classification, gene structure and evolutionary characteristics, we explored the evolutionary characteristics and functional division mechanism of the family. Combined with data such as subcellular localization and protein interaction, we elucidated the adaptive relationship between expression patterns and physiological demands, and simultaneously screened magnesium breeding-related genetic loci such as single nucleotide polymorphism (SNP) variations of *TaMRS2* genes. Finally, we integrated multi-dimensional data to clarify the magnesium transport regulatory mode, thereby defining its mechanism of action in photosynthesis and reproductive development. This research not only systematically clarifies the characteristics and evolutionary rules of the wheat magnesium transporter family, reveals the core regulatory logic of evolution-function-expression-variation, enriches the molecular mechanism of plant magnesium nutrition regulation, but also offers precise support for molecular breeding of magnesium-efficient wheat by identifying key functional genes and SNP targets. It is of great significance for improving wheat stress adaptability and yield stability, safeguarding food security, and promoting the green development of agriculture.

## Materials and methods

### Identification and classification analysis of magnesium transporter genes in wheat

To investigate the genome-wide magnesium transporter gene family members in wheat, the Chinese spring wheat genome sequence was downloaded from the EnsemblPlants website (http://plants.ensembl.org/index.html). A local genome database was constructed using the completed genome, CDS, and protein sequences. The HMM profile related to the MGT domains including three types of NIPA (PF05653), CorA (PF01544) and MRS2/MGT (with two CorA-like domain) was retrieved using the Pfam database (Mistry et al., 2021). The MRS2/CorA/NIPA protein sequences from *A. thaliana* and *O. sativa* (**Supplementary Table 1**) were used as query sequences to search against the wheat protein dataset using the BLASTP program (https://blast.ncbi.nlm.nih.gov). The default parameters were adopted with a threshold of E-value *<* 1e^-5^. The NCBI-Batch CD-Search (Marchler-Bauer et al., 2017) (https://www.ncbi.nlm.nih.gov/Structure/bwrpsb/bwrpsb.cgi), Pfam database (http://pfam.xfam.org/) and SMART database (http://smart.embl.de/) were used to further confirm the candidate *MRS2/CorA/NIPA* genes of *T. aestivum.* There were other spliced transcripts in the candidate genes of these species, and we selected the first splice variant as a representative for subsequent analysis.

The molecular weight (MW), instability index (II), theoretical isoelectric point (pI), aliphatic index (AI), and grand average hydrophobicity (GRAVY) of the *MRS2/CorA/NIPA* genes were computed using the ExPASy server (Duvaud et al., 2021). Plant-mPLoc (Chou et al., 2012) (http://www.csbio.sjtu.edu.cn/bioinf/plant-multi/) and BUSCA (Savojardo et al., 2018) (Bologna Unified Subcellular Component Annotator, http://busca.biocomp.unibo.it) were used to predict the subcellular localization of the MRS2/CorA/NIPA proteins.

### Phylogenetic analyses of magnesium transporter genes in wheat

To explore the evolutional relationships of the magnesium transporter gene family members in different species. We built two trees. In the first tree, a total of 114 magnesium transporter proteins, including 4 AtCorAs, 9 TaCorAs, 7 AtNIPAs, 8 OsNIPAs, 30 TaNIPAs, 11 AtMRS2s, 9 OsMRS2s, 12 ZmMRS2s, 24 TaMRS2s were included in the phylogenetic analysis. In the second tree, a total of 116 MRS2 proteins, including five monocotyledons (*O. sativa, Z. mays, T.aestivum, S.* sp*ontaneum, T. turgidum*) and three dicotyledons (*A. thaliana, S. lycopersicum, Bn.distachyon*), were included in the phylogenetic analysis. Then, evolutionary analyses were conducted in MEGA X (Kumar et al., 2018) (Molecular Evolutionary Genetics Analysis) software using the neighbor-joining meth. The percentage of replicate trees in which the associated taxa clustered together in the bootstrap test (1000 replicates) is shown next to the branches. The trees were visualized and optimized in iTOL (Letunic and Bork, 2024). Jalview 2.11 software (http://www.jalview.org/) with the Mafft method with default parameters was utilized to conduct multiple sequence alignment.

### Chromosome distribution and synteny analysis of magnesium transporter genes in wheat

\To determine the chromosomal distribution of the magnesium transporter family genes, the illustration of these genes was visualized on their specific chromosomal location in the genome annotation using TBtools-II software (Chen et al., 2023). The syntenic blocks information of the magnesium transporter family genes, as well as those between wheat and 6 species (three monocotyledons and three dicotyledons), were conducted with MCScanX software (http://bdx-consulting.com/mcscanx-protocol/) (Wang et al., 2024) using the chromosomal location data and protein sequences of the TaMRS2s/CorAs/NIPAs, and the chromosomal location and synteny analysis diagrams were visualized using TBtools-II software (Chen et al., 2023). Gene duplication events of *TaMRS2s/CorAs/NIPAs* and synteny relationships between the aforementioned species were visualized using TBtools-II. A ratio of Ka/Ks > 1 indicated positive selection; Ka/Ks = 1 indicated neutral selection, and Ka/Ks < 1 indicated negative selection. Subsequently, the divergence time of collinear gene pairs was calculated using the duplication events formula T = Ks/(2λ × 10^-6^) in millions of years (Mya), with λ = 6.5 × 10^-9^ (Ge et al., 2022).

### Gene structure and cis-acting element analysis of *TaMRS2* genes

Intron/exon information of *MRS2/CorA/NIPA* genes were extracted from the genome annotation file and submitted to the Gene Structure Display Server (GSDS, https://gsds.gao-lab.org/Gsds_help.php) to display the gene structures. The MEME program was used to identify motifs in *MRS2/CorA/NIPA* genes sequence with the following parameters: 15 motifs with an optimal motif width of 6–100 amino acid residues and any number of repeats (Bailey et al., 2009). The gene structures of the *TaMRS2* were drawn using the TBtools-II software. Upstream regions (2000 bp) of *MRS2/CorA/NIPA* genes from the transcription start site were extracted. Cis-acting elements were analyzed in the PlantCARE database (Ge et al., 2022).

### Protein–protein interaction network analysis in *TaMRS2* family genes

All the predicted MRS2/CorA/NIPA proteins were submitted to the STRING database (https://string-db.org/cgi/input.pl). The minimum required interaction score was set to high confidence (0.700). Next, we performed a biological process (Gene Ontology) enrichment analysis on the proteins in the interaction network, using the default parameters from the STRING database.

### Expression analysis of *TaMRS2* genes

In order to search the tissue-specific expression of MRS2/CorA/NIPA protein in wheat, Transcriptional data for *MRS2/CorA/NIPA* genes were obtained from the wheat expression website (http://www.wheat-expression.com/download) (Ramírez-González et al., 2018; Borrill et al., 2016) and were used to explore the potential biological functions of *TaMRS2* genes in growth and development. Systematic clustering analysis was performed based on the log2 of transcripts per million (TPM) values for the *MRS2/CorA/NIPA* genes. OmicStudio (https://www.omicstudio.cn/tool) was used to display the expression patterns in a heat map. Using BAR online expression analysis, create an “electronic fluorescence pictogram” representation of the target *TaMRS2-10, TaMRS2-14, TaMRS2–16* and *TaMRS2–19* genes expression pattern based on the Wheat Atlas dataset.

### Quantitative real-time PCR analyses of *TaMRS2* genes in response to environmental stresses

In this study, the seeds of the hexaploid common wheat variety “Zhengmai 7698” which is a wheat variety with a large planting area in the Huanghuai Wheat Region of China, were surface-sterilized with 2% hydrogen peroxide, rinsed thoroughly with distilled water, and germinated with water saturation at 25 °C for 2 days in Petri dishes on three layers of filter paper. The young seedlings were transformed and grown in 1/2 Hoagland’s culture solution under a 14 h light (25 °C)/10 h dark (20 °C) photoperiod. When the wheat grew to two leaves and one heart, the plants were subsequently treated with 16% polyethylene glycol 6000 (PEG6000). For cold stress, rice seedlings were exposed to 4 °C for 12 h. For heat stress, rice seedlings were exposed to 40 °C for 12 h. New leaves of the three seedlings were collected as biological replicates, and each treatment had three replicates.

Total RNA was extracted using RNAiso Plus (Code No. 9108; TaKaRa, Beijing, China) and cDNA was synthesized using the PrimeScript™ RT Master Mix (Code No. RR036A; TaKaRa, Beijing, China). Quantitative real-time PCR was performed using the CFX Touch™ Real-Time PCR Detection System (Bio-Rad Laboratories, Hercules, CA, USA) and the SG Fast qPCR Master Mix (B639271-0001; Sangon Biotech, Shanghai, China). Relative expression levels were determined using the 2^(-ΔΔCt)^ method, and β-actin was used as the internal control to normalize the expression levels of *TaMRS2* genes. Specific primers used for qRT-PCR are listed in **Supplementary Table 2**.

### SNP analysis of TaMRS2-13, TaMRS2-14, TaMRS2-19, and TaMRS2-20

Within the Wheat-SnpHub-Portal, a collaborative database for wheat genomic variation, SNP analysis was conducted on sequences spanning 3000 base pairs upstream and downstream of the genes *TaMRS2-13, TaMRS2-14, TaMRS2-19*, and *TaMRS2-20*. Locations exhibiting significant nucleotide variation were then subjected to a visualization analysis of their global distribution. The results exported as default raw images.

### Determination of subcellular localization of nine TaMRS2s

Full-length open reading frames of *TaMRS2-1, TaMRS2-2*, *TaMRS2-3*, *TaMRS2-16, TaMRS2-17*, *TaMRS2-18, TaMRS2-19, TaMRS2-20*, and *TaMRS2–21* were obtained from “zhengmai 7698” cDNA (**Supplementary Table 2**). The CDS sequences of *TaMRS2-1, TaMRS2-2*, *TaMRS2-3*, *TaMRS2-16, TaMRS2-17*, *TaMRS2-18, TaMRS2-19, TaMRS2-20*, and *TaMRS2–21* were cloned into the pJIT16318 vector at the BamHI site using specific primers (**Supplementary Table 2**). The pJIT16318 vector contained a CaMV 35S promoter and C-terminal GFP. Transient expression assays were conducted as described by Hao*, et al.* (Hao et al., 2025). Approximately 4 × 10^4^ mesophyll protoplasts were isolated from 12-day-old wheat seedlings. The transfected protoplasts were incubated at 23 °C for 12 h. GFP fluorescence in the transformed protoplasts was imaged using a confocal laser-scanning microscope (LSM 700; Zeiss).

## Results

### Identification and classification analysis of *MRS2/CorA/NIPA* genes in wheat

Twenty-four *MRS2* genes, nine *CorA* genes and thirty *NIPA* genes were identified in wheat based on search of the NCBI-Batch CD-Search, Pfam database and SMART database (**Table 1**). These *MRS2/CorA/NIPA* genes were named *TaMRS2–1* to *TaMRS2*-*24*, *TaCorA*-*1* to *TaCorA-9*, and *TaNIPA*-*1* to *TaNIPA-30* according to their positions on the chromosomes. The lengths of the TaMRS2 encoded proteins ranged from 348 to 468 amino acids. The Mw of TaMRS2 proteins ranged from 39688.7 Da to 51164.41 Da, while the pI values were between 4.74 and 6.43. The GRAVY of TaMRS2 proteins in wheat varied from 0.082 to -0.358, indicating that they were all hydrophilic proteins, except for *TaMRS2-22/23* (**Table 1**). We used two methods (Plant-mPLoc and BUSCA) to predict the subcellular localization of the TaMRS2 proteins. The results showed that the 24 TaMRS2 proteins may be localized in the nucleus, chloroplast or cytoplasm, and most members were predicted to be located in the membrane system (**Table 1**).

**Table 1:** Detailed properties of identified *TaMRS2/CorA/NIPA* genes.

ID	Name	pI	MW	CDS	Length	I. I.	GRAVY	Sub.	
								BUSCA	Plant-mPLoc
TraesCS1A02G205400.1	*TaMRS2-1*	6.43	50775.05	1395	464	54.84	-0.167	organelle membrane	Chloroplast.
TraesCS1B02G219000.1	*TaMRS2-2*	6.23	51164.41	1407	468	55.34	-0.162	organelle membrane	Chloroplast.
TraesCS1D02G208700.1	*TaMRS2-3*	6.23	50894.13	1398	465	53.96	-0.166	organelle membrane	Chloroplast.
TraesCS2A02G354100.1	*TaMRS2-4*	5.17	47298.31	1287	428	49.91	-0.048	endomembrane system	Chloroplast. Nucleus.
TraesCS2B02G375200.1	*TaMRS2-5*	5.24	47437.51	1287	428	49.93	-0.064	endomembrane system	Chloroplast. Nucleus.
TraesCS2D02G354900.1	*TaMRS2-6*	5.56	45456.26	1239	412	49.85	-0.069	endomembrane system	Chloroplast.
TraesCS3A02G380500.1	*TaMRS2-7*	5.33	45518.86	1227	408	45.03	-0.307	endomembrane system	Chloroplast. Nucleus.
TraesCS3B02G413100.1	*TaMRS2-8*	5.58	45214.62	1224	407	43.68	-0.295	endomembrane system	Chloroplast. Nucleus.
TraesCS3D02G373600.1	*TaMRS2-9*	5.26	45407.67	1224	407	42.69	-0.316	plasma membrane	Chloroplast. Nucleus.
TraesCS3A02G380600.1	*TaMRS2-10*	4.97	45290.65	1218	405	45.48	-0.351	endomembrane system	Chloroplast. Cytoplasm. Nucleus.
TraesCS3B02G413200.1	*TaMRS2-11*	4.92	45207.46	1218	405	44.65	-0.324	endomembrane system	Nucleus.
TraesCS3D02G373700.1	*TaMRS2-12*	4.9	45395.67	1218	405	46.75	-0.358	endomembrane system	Chloroplast. Cytoplasm. Nucleus.
TraesCS3A02G414000.1	*TaMRS2-13*	4.86	49332.86	1356	451	44.76	-0.22	endomembrane system	Nucleus.
TraesCS3B02G448800.1	*TaMRS2-14*	4.86	49127.73	1350	449	44.36	-0.191	endomembrane system	Chloroplast.
TraesCS3D02G408500.1	*TaMRS2-15*	4.86	49246.81	1353	450	44.29	-0.208	endomembrane system	Chloroplast. Nucleus.
TraesCS4A02G286400.1	*TaMRS2-16*	5.43	49578.14	1347	448	57.92	-0.144	mitochondrial membrane	Nucleus.
TraesCS4B02G027800.1	*TaMRS2-17*	5.64	49639.31	1347	448	56.78	-0.151	organelle membrane	Chloroplast. Nucleus.
TraesCS4D02G025300.1	*TaMRS2-18*	5.71	49671.36	1344	447	57.78	-0.161	mitochondrial membrane	Chloroplast. Cytoplasm. Nucleus.
TraesCS5A02G382400.1	*TaMRS2-19*	4.85	43092.65	1179	392	40.17	-0.139	endomembrane system	Chloroplast. Nucleus.
TraesCS5B02G386300.1	*TaMRS2-20*	XXXX		1167	388	39.78	-0.161	endomembrane system	Nucleus.
TraesCS5D02G391700.1	*TaMRS2-21*	4.85	42764.28	1167	388	39.98	-0.158	endomembrane system	Chloroplast. Nucleus.
TraesCS7A02G506600.1	*TaMRS2-22*	5.18	49161.28	1332	443	50.08	0.001	endomembrane system	Chloroplast.
TraesCS7B02G414200.1	*TaMRS2-23*	4.74	39688.7	1047	348	50.65	0.082	plasma membrane	Nucleus.
TraesCS7D02G494700.1	*TaMRS2-24*	5.18	49444.61	1338	445	50.82	-0.002	endomembrane system	Chloroplast.
TraesCS3A02G215500.1	*TaCorA1*	6.12	63755.08	1719	572	45.91	-0.118	plasma membrane	Chloroplast
TraesCS3B02G245500.1	*TaCorA2*	5.89	63842.12	1719	572	47.63	-0.107	plasma membrane	Chloroplast
TraesCS3D02G217500.1	*TaCorA3*	6.04	63707.04	1716	571	47.45	-0.113	plasma membrane	Chloroplast
TraesCS3A02G241600.1	*TaCorA4*	6.48	61604.43	1665	554	46.28	-0.183	endomembrane system	Chloroplast
TraesCS3B02G269800.1	*TaCorA5*	6.4	61623.34	1665	554	45.44	-0.196	endomembrane system	Chloroplast
TraesCS3D02G241800.2	*TaCorA6*	6.4	61367.08	1659	552	45.44	-0.191	endomembrane system	Chloroplast
TraesCS7A02G310600.1	*TaCorA7*	6.86	51881.93	1449	482	45.95	0.081	plasma membrane	Chloroplast
TraesCS7B02G210400.1	*TaCorA8*	6.86	51775.79	1449	482	47.61	0.083	plasma membrane	Chloroplast
TraesCS7D02G307100.2	*TaCorA9*	7.35	51794.78	1449	482	44.57	0.071	plasma membrane	Chloroplast
TraesCS1A02G264700.1	*TaNIPA1*	8.51	41314.59	1149	382	33.56	0.567	plasma membrane	Cell membrane
TraesCS1B02G275400.1	*TaNIPA2*	6.08	33364.13	924	307	39.67	0.588	endomembrane system	Cell membrane
TraesCS1D02G264900.1	*TaNIPA3*	8.72	41455.75	1149	382	35.04	0.55	plasma membrane	Cell membrane
TraesCS1A02G333200.1	*TaNIPA4*	6.24	39097.96	1089	362	41.07	0.62	plasma membrane	Cell membrane
TraesCS1B02G346700.1	*TaNIPA5*	6.24	39071.93	1086	361	40.82	0.617	plasma membrane	Cell membrane
TraesCS1D02G335900.1	*TaNIPA6*	6.13	38910.72	1080	359	38.17	0.62	plasma membrane	Cell membrane
TraesCS2A02G278100.1	*TaNIPA7*	6.14	34801.01	957	318	29.07	0.76	endomembrane system	Cell membrane
TraesCS2B02G295700.1	*TaNIPA8*	6.37	34543.66	951	316	26.36	0.732	endomembrane system	Cell membrane
TraesCS2D02G277100.1	*TaNIPA9*	6.14	34791.03	957	318	26.26	0.757	endomembrane system	Cell membrane
TraesCS2A02G557600.1	*TaNIPA10*	8.44	39086.74	1095	364	34.09	0.585	plasma membrane	Cell membrane
TraesCS2B02G617600.1	*TaNIPA11*	8.44	38998.71	1089	362	34.16	0.596	plasma membrane	Cell membrane
TraesCS2D02G567600.1	*TaNIPA12*	8.67	45490.23	1275	424	35.95	0.425	endomembrane system	Cell membrane
TraesCS3A02G383600.1	*TaNIPA13*	7.52	41514.85	1170	389	38.15	0.644	endomembrane system	Cell membrane
TraesCS3B02G416000.1	*TaNIPA14*	6.42	38648.48	1086	361	33.15	0.67	endomembrane system	Cell membrane
TraesCS3D02G376700.1	*TaNIPA15*	6.87	38611.52	1083	360	35	0.693	endomembrane system	Cell membrane
TraesCS3A02G407400.1	*TaNIPA16*	6.52	39072.91	1095	364	33.42	0.608	endomembrane system	Cell membrane
TraesCS3B02G440800.1	*TaNIPA17*	6.52	39060.92	1095	364	33.12	0.608	endomembrane system	Cell membrane
TraesCS3D02G402700.1	*TaNIPA18*	6.52	39084.97	1095	364	33.42	0.624	endomembrane system	Cell membrane
TraesCS4A02G215000.1	*TaNIPA19*	6.65	40287.15	1122	373	38.3	0.544	endomembrane system	Cell membrane
TraesCS4B02G101200.1	*TaNIPA20*	6.37	40259.05	1122	373	39.05	0.545	endomembrane system	Cell membrane
TraesCS4D02G097900.2	*TaNIPA21*	6.37	40273.08	1122	373	39.05	0.545	endomembrane system	Cell membrane
TraesCS6A02G189200.1	*TaNIPA22*	8.87	35575.13	987	328	34.05	0.71	endomembrane system	Cell membrane
TraesCS6B02G215400.1	*TaNIPA23*	8.87	35478.01	984	327	33.53	0.717	endomembrane system	Cell membrane
TraesCS6D02G177800.1	*TaNIPA24*	8.99	35308.83	981	326	31.52	0.733	endomembrane system	Cell membrane
TraesCS6A02G194800.1	*TaNIPA25*	9.16	37821.34	1005	334	40.64	0.534	endomembrane system	Cell membrane
TraesCS6B02G220700.1	*TaNIPA26*	8.35	41609.67	1122	373	38.8	0.462	plasma membrane	Cell membrane
TraesCS6D02G182700.1	*TaNIPA27*	8.65	41814.91	1125	374	42.24	0.435	plasma membrane	Cell membrane
TraesCS7A02G527900.1	*TaNIPA28*	XXXX		1032	343	33.06	0.737	endomembrane system	Cell membrane
TraesCS7B02G444600.1	*TaNIPA29*	5.94	36362.78	1032	343	34.92	0.702	endomembrane system	Cell membrane
TraesCS7D02G515800.1	*TaNIPA30*	5.94	36304.7	1032	343	34.92	0.71	endomembrane system	Cell membrane

Chr, Chromosomal Location; Length, Amino acid length; aa; CDS, length of coding DNA sequence; bp; pI, Isoelectric point; MW, Molecular weight, KD; I. I., Instability index; A. I., Aliphatic index; GRAVY, Grand average of hydropathy; Sub., Subcellular localization; Nuc., Nucleus; Chl., Chloroplast.

The results showed that the TaCorA proteins is longer than the TaMRS2 proteins, while the TaNIPA proteins is shorter than the TaMRS2 proteins. The Mw of TaNIPA proteins ranged from 33364.13 Da to 45490.23 Da. The GRAVY of TaNIPA proteins in wheat varied from 0.425 to 0.757. All TaNIPA proteins are hydrophobic proteins (**Table 1**). The results showed that the 30 TaNIPA proteins may be localized in the plasma membrane, endomembrane system or cell membrane. However, TaCorAs were predicted to be located in the plasma membrane, endomembrane system or chloroplast (**Table 1**).

### Phylogenetic analysis and multiple sequence alignment of magnesium transporter genes

**Figure 1:**
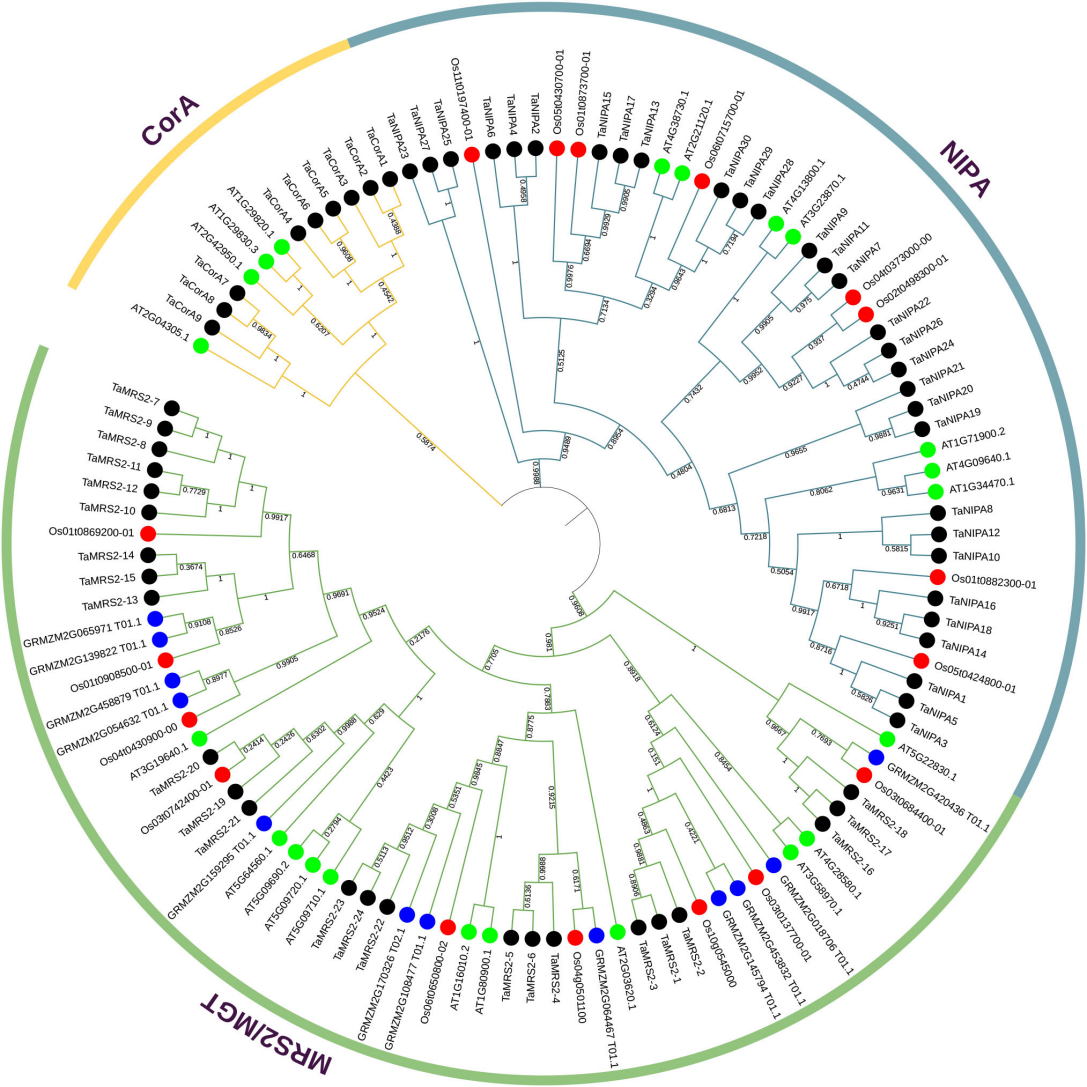
Phylogenetic tree of magnesium transporter genes in wheat, rice, maize and *Arabidopsis*. The tree was analyzed in MEGA X by using the neighbor-joining method. The magnesium transporter genes from wheat, rice, maize and *Arabidopsis* are distinguished with black, red, blue and green dots. The MGT proteins were grouped into three distinct clades (MRS2, CorA and NIPA), which are indicated by colored branches.

**Figure 2:**
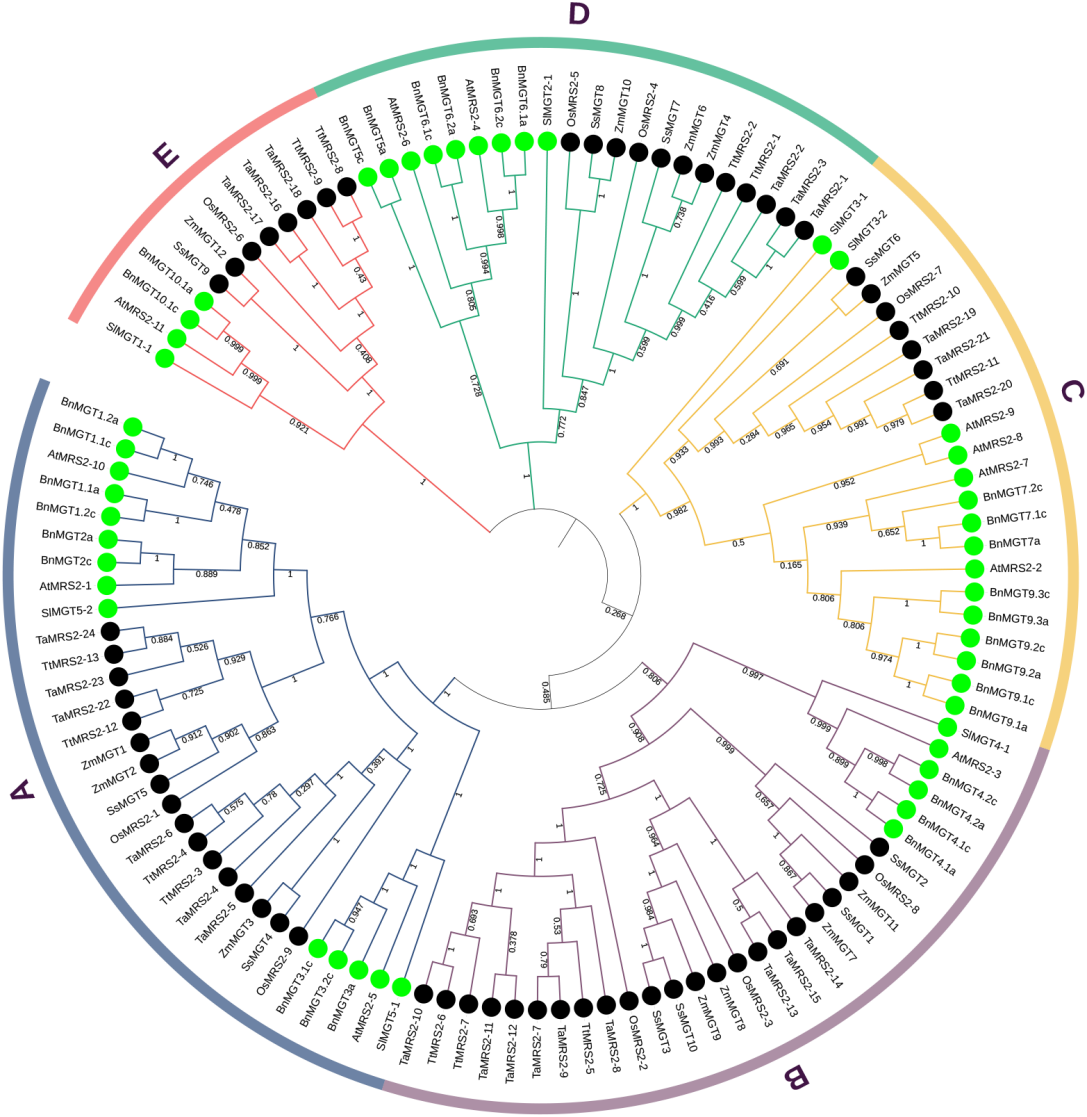
Phylogenetic tree of *MRS2* genes in five monocotyledons (*O. sativa, Z. mays, T.aestivum, S.* sp*ontaneum, T. turgidum*) and three dicotyledons (*A. thaliana, S. lycopersicum, Bn.distachyon*). The tree was analyzed in MEGA X by using the neighbor-joining method. The MRS2s monocotyledons and dicotyledons are distinguished with black and green dots. The MRS2 proteins were grouped into five distinct clades (clades A-E), which are indicated by colored branches.

To evaluate the evolutionary relationships of magnesium transporter genes in wheat and across species, two neighbor-joining phylogenetic tree was constructed using full-length MRS2 proteins (**Figures 1**, **2**). The first tree contains *MRS2/CorA/NIPA* family genes that have been identified in four species, including *wheat*, *A. thaliana, rice and maize* (**Figure 1**). In the result, it’s not hard to find that the *MRS2* genes can be distinctly differentiated from the CorA-like and NIPA-like gene family. And everyone was present in distinct and separate clades in the phylogenetic tree. To further analyze the evolutionary relationship of *MRS2* gene family in monocotyledons and dicotyledons, five monocotyledons and three dicotyledons *MRS2* genes were used to build the second evolutionary tree (**Figure 2**). Phylogenetic analysis showed that *MRS2* family proteins can be divided into five clades (clades A to E). Each species has members in these five clades. Clade A was the largest, with 31 *MRS2* members, and clade E was the smallest, with only 12 members.

Multiple sequence alignments of TaMRS2 and OsMRS2 domains were performed (**Supplementary Figure 1**). All the MRS2 proteins have two TM domains at the C-terminal region according to the multiple sequence analysis. The first TM domain is about 29 aa and most comprised a conserved GMN motif. Interestingly, previous studies have shown that two *MRS2/MGT* genes (*AtMRS2-8* and *AtMRS2-9*) in A. thaliana are known as pseudogenes that lack their GMN conserved motif in their sequence. Mutation of the conserved GMN motif was reported in which either AMN or GIN motif is formed. The AMN variant was reported in both O. sativa and Z. mays, where GIN lies in the single *AtMRS2-8* gene of *O. sativa* (Saito et al., 2013; Li et al., 2016). Our result show that this motif was altered to AMN in TaMRS2-1, TaMRS2–2 and TaMRS2-3 (**Supplementary Figure 1**), these alterations were proposed to be associated with cation selectivity (Sponder et al., 2013).

### Chromosomal location, gene duplication, and synteny analysis of magnesium transporter genes

**Figure 3:**
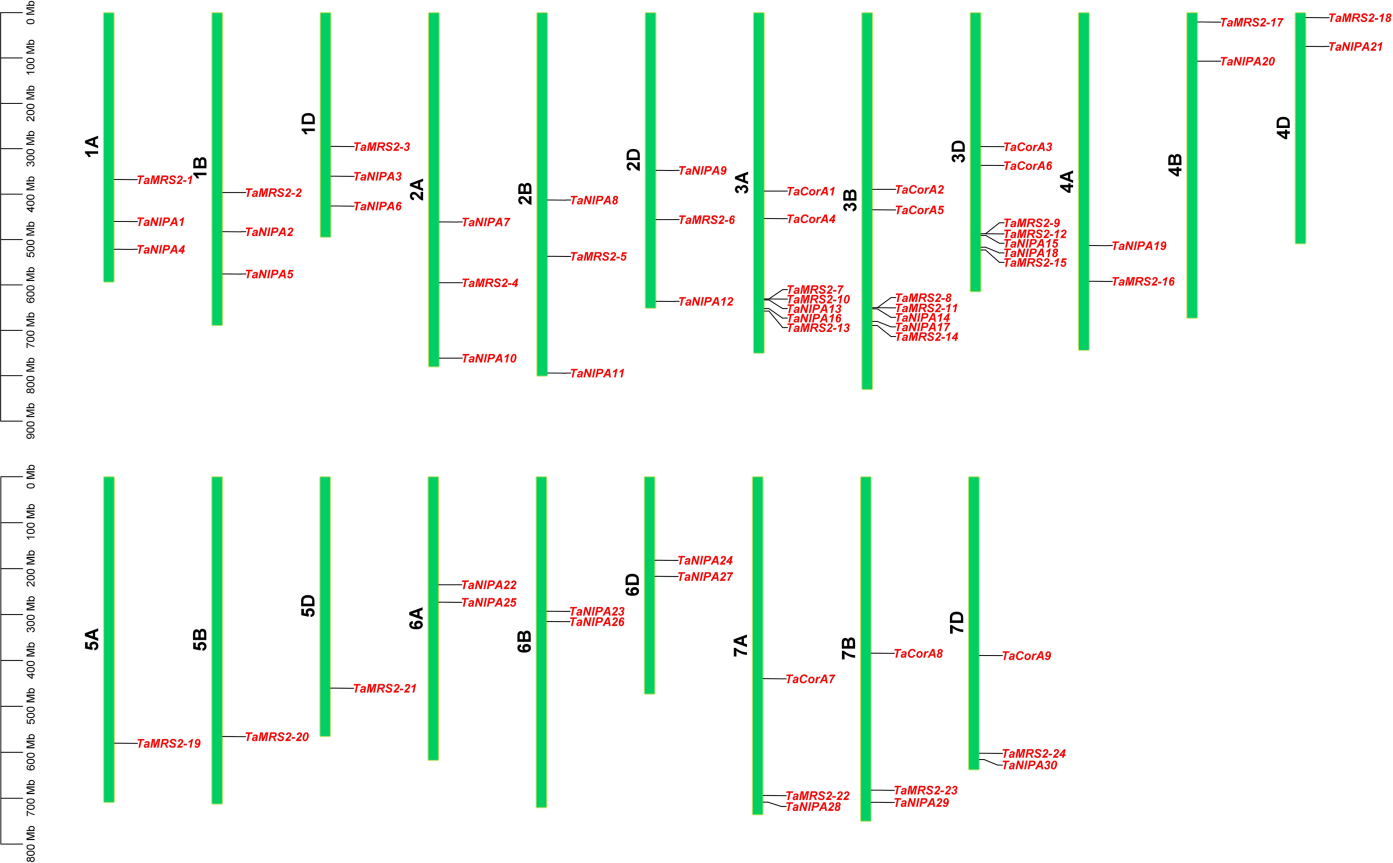
Chromosomal localization of the magnesium transporter genes. The light green column represents the chromosome. The depth of blue in the columns represent the density of genes on the chromosome.

Based on the reference GFF3 files, the physical positions of *TaMRS2/CorA/NIPA* genes on the corresponding chromosomes are shown in **Figure 3**. The identified *TaMRS2/CorA/NIPA* genes could be mapped on every chromosome and evenly across the three sub-genomes. The map shows that chromosomes 3A/3B/3D harbor the largest number of *TaMRS2/CorA/NIPA* genes (21), whereas chromosome 5A/5B/5D contains the least (3).

**Figure 4:**
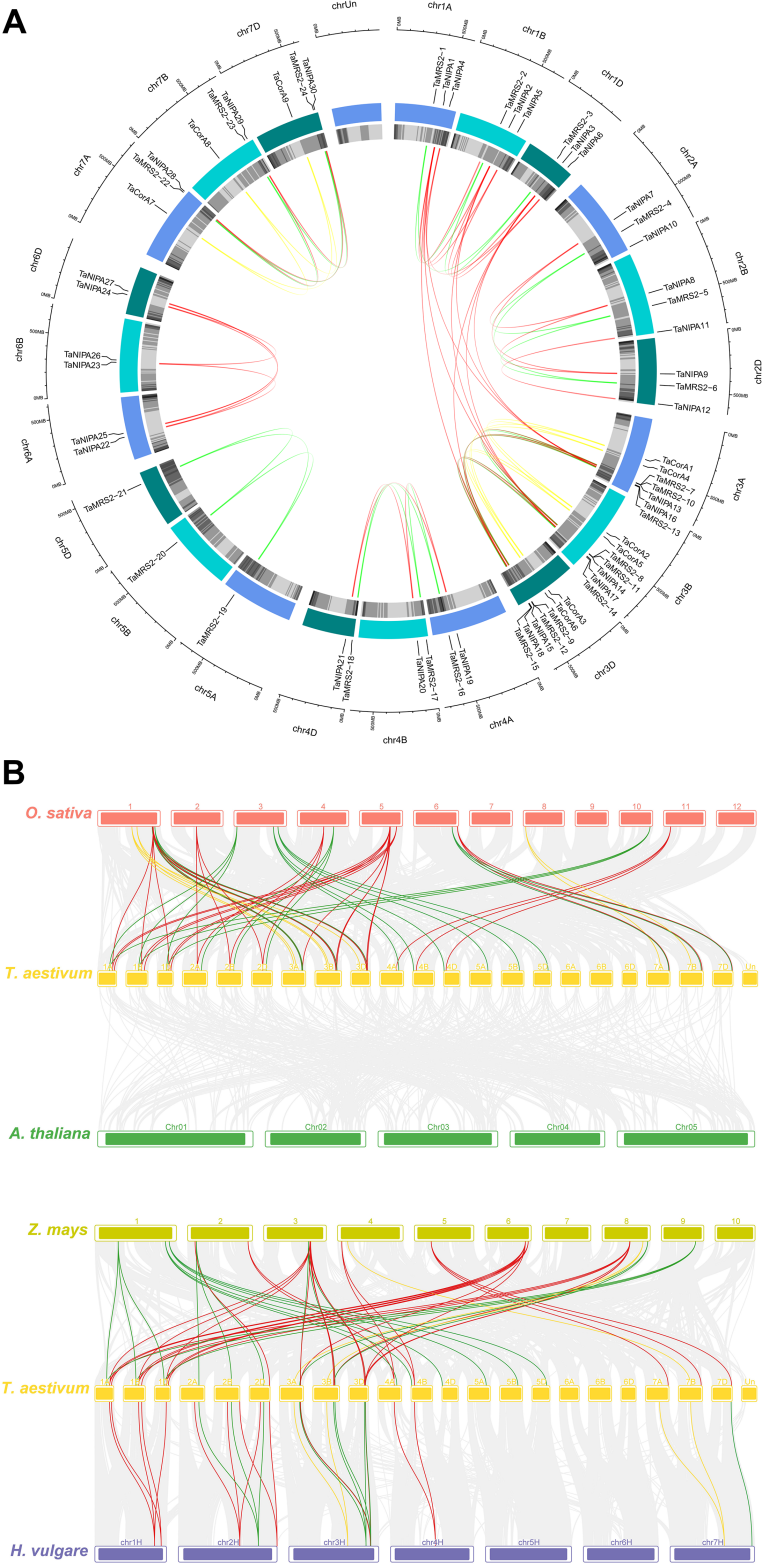
Synteny analysis of magnesium transporter genes. **(A)** Synteny analysis of magnesium transporter genes in wheat. All genes were mapped to their respective locus in the wheat genome in a circular diagram using shinyCircos. Subgenomes are indicated by different shades of blue (outer track), and chromosomal segments are indicated by shades of gray (inner track). Homoeologous magnesium transporter genes were inferred by phylogeny (for details see the Materials and Methods section) and linked with chromosome-specific colors. **(B)** Synteny analysis of magnesium transporter genes between wheat and four representative plants (maize, barley, rice and *Arabidopsis*). Each different species is replaced with a different color. The gray line in the background indicates a collinear block in the genome of wheat and other plants, while the line highlights the isomorphic magnesium transporter gene pair. Homoeologous magnesium transporter genes were inferred by phylogeny (for details see the Materials and Methods section) and linked with chromosome-specific colors.

Gene duplication is an indispensable mechanism by which organisms create new genes with similar or different functions (Song et al., 2013). Therefore, we analyzed the duplication events that occurred in the magnesium transporter gene family (**Figure 4**). Ka/Ks, the non-synonymous substitution ratio, determines the selection pressure for duplicated genes. According to the results (**Supplementary Table 1**), only very few *TaMRS2* gene pairs had Ka/Ks ratios >1, suggesting that the evolution of *TaMRS2* genes was accompanied by strong purifying selection. The Ka/Ks ratios between wheat and three monocotyledonous plants were calculated based on the collinear gene pairs. Except for very few genes, the values of the other collinear gene pairs were all below 1, which confirmed that the evolution of the wheat *MRS2* gene family underwent strong purifying selection. However, the Ka/Ks ratios of the collinear gene pairs between wheat and the three dicots could not be calculated properly. This is because most synonymous mutation sites have synonymous mutations; that is, the degree of sequence divergence and evolutionary distance is too large. Some *TaMRS2* genes have formed at least five homologous gene pairs, such as *TaMRS29*, which may have played key roles in the evolution of the *MRS2* gene family (**Figure 4**; **Supplementary Table 1**).

### Gene structure and promoter cis-element analysis of magnesium transporter genes

**Figure 5:**
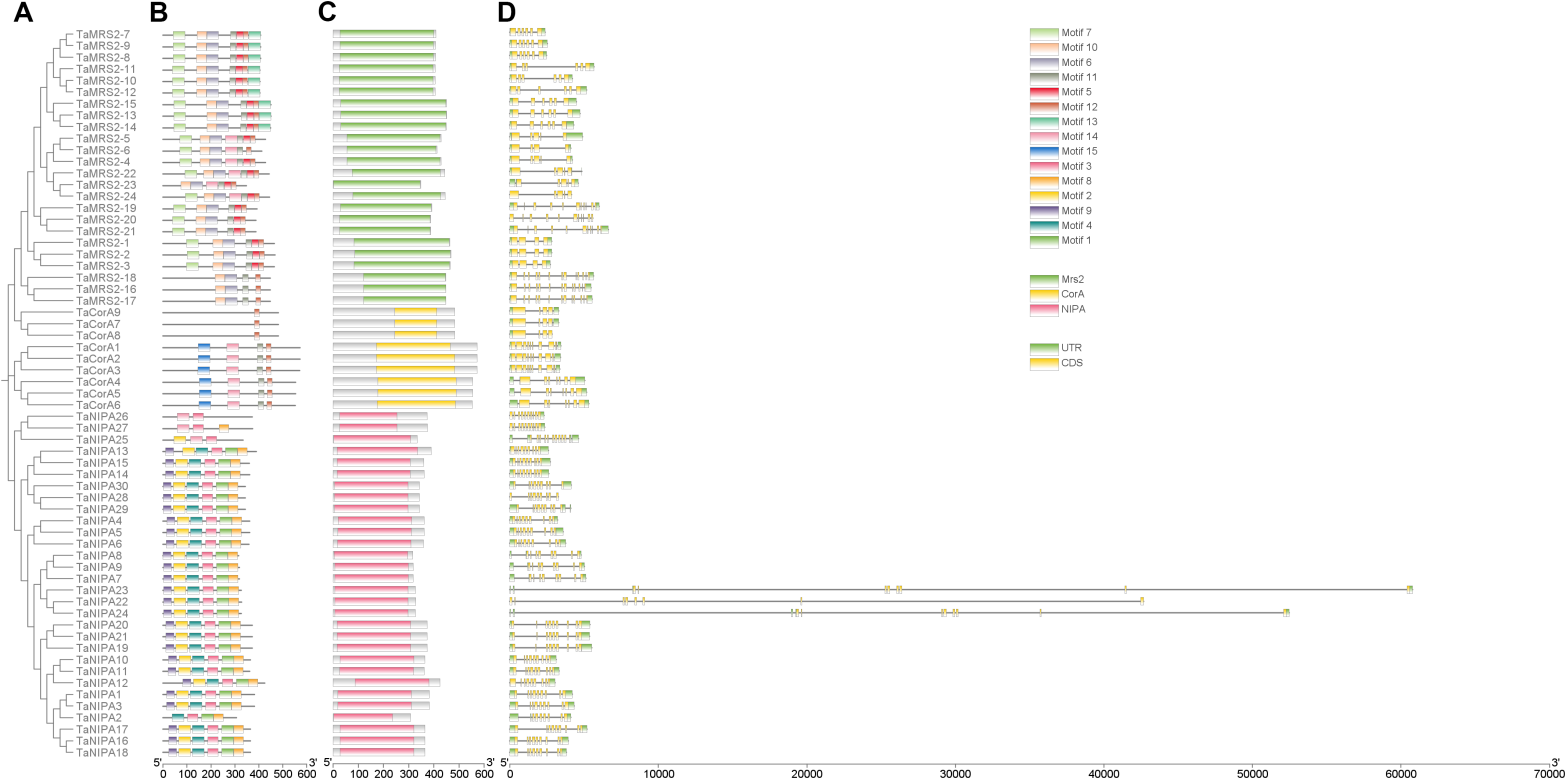
Comparative analysis of the phylogenetics, conserved motifs, exon-intron structure, and protein structure of MGT family in wheat. **(A)** The phylogenetic tree of 63 TaMGT proteins were constructed by using MEGA X; **(B)** Motif composition models of 63 TaMGT proteins. Different motifs are color-coded. **(C)** Schematic representation of functional domains of TaMGTs. Bioinformatics analysis of functional conserved domains were performed by Pfam database (http://pfam.janelia.org/); **(D)** GSDS2.0 software was employed to generate the gene structure of 63 TaMGT proteins. Untranslated regions (UTRs) are indicated by green boxes; exons are indicated by yellow boxes; introns are indicated by black lines.

Further analysis of the gene structure and the conserved domains in *TaMRS2/CorA/NIPA* genes are shown in **Figure 5**. We constructed a phylogenetic tree of *TaMRS2/CorA/NIPA* genes and analyzed the conserved motifs, domains, exons and introns (**Figure 5**). The results of gene structure analysis indicate that the *TaMRS2/NIPA* genes have relatively complex structure. The TaMRS2 proteins exhibit similar conserved motif compositions, in particular, all TaMRS2 proteins except TaMRS2-16/17/18 contain motif 7, motif 6, motif 10, motif 11 and motif 12, suggesting that these five motifs are important components for TaMRS2 protein sequences. Meanwhile, MRS2 members in separate subgroups also contain their specific motifs. For example, motif 4 is present in subfamily B; motif 7 is not found in subfamily E. The NIPA conserved motif is very different from MRS2/CorA. The TaNIPA proteins exhibit similar conserved motif compositions, in particular, all TaNIPA proteins except TaNIPA-2/25/26/27 contain motif 1, motif 2, motif 3, motif 4, motif 8 and motif 9 suggesting that these six motifs are important components for TaNIPA protein sequences. The results of gene structure analysis indicate that the *TaCorA* genes has relatively simple structure. All TaCorA protein contain motif 12. The results show that TaCorA proteins and TaMRS2 proteins are similar in structure.

Protein domains are often functional carriers. We can find out TaMRS2/CorA/NIPA proteins only have one domain (**Figure 5**) And the domain in TaMRS2s are close to C-terminal region, the domain in TaCorAs is in the middle regio, the domain in TaNIPAs are close to N-terminal region. This may indicate that the TaMRS2/CorA/NIPA proteins are exercising its function in different region. To resolve the detailed structure of *TaMRS2* genes, we also visualized its introns, exons, and UTRs (**Figure 5**). The result shows that despite being different with regard to exon position, *TaMRS2* genes from the same subfamily share a similar genetic structure, and the number of exons in most TaMRS2 genes is not conserved. *TaMRS2-1/2/3* and *TaCorA-7/8/9* have four introns which is the fewest exons in all subfamily. And *TaMRS2-16/17/18* have thirteen exons which is the most. *TaNIPAs* have eight-eleven exons which is the most one has a lot of tiny exons in all subfamily. In short, members in the same subfamily share similar gene structure and motif compositions, while different subgroups contain the specific structure, implying that the magnesium transporter gene family presents the functional conservation and diversity during evolution.

**Figure 6:**
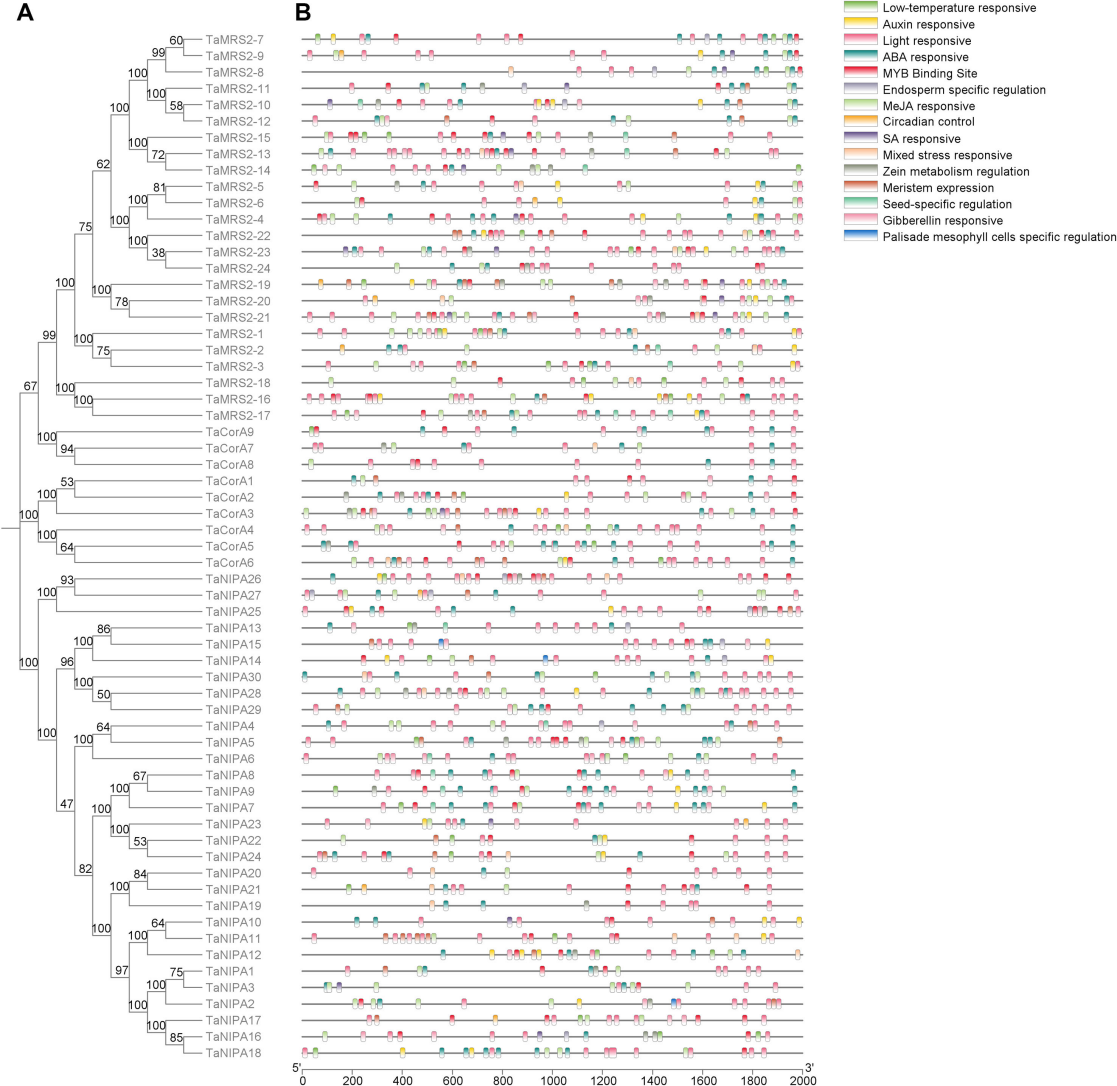
Predicted cis-elements in the promoter regions of the wheat *MGT* genes. **(A)** Comparative analysis of the phylogenetics of *MGT* family in wheat; **(B)** Cis-acting elements involved in stresses in the promoter of *TaMGT* genes. All elements in the promoter of TaMGT genes were shown in **Supplementary Table 2**. The different colors of the grid indicated different promoter elements in these *MGT* genes.

The cis-acting regulatory element is a specific motif that binds to appropriate transcription factor to control gene transcription (Hernandez-Garcia and Finer, 2014). In order to study the response of magnesium transporter genes to various signal factors, the sequence of 2000 bp upstream of its transcription start position was used to find various cis-acting elements. A totally 15 categories cis-acting elements were predicted (**Figure 6**; **Supplementary Table 2**). These cis-acting elements were related to environmental stress, hormone response, development, light response, site binding and other functions (**Figure 6**). The most abundant elements were light-responsive elements, including G-box, GT1-motif and GATA-motif. 5 hormone responsive elements were identified and these are mainly involved in response to abscisic acid (ABA) or methyl jasmonate (MeJA) (**Figure 6**). Among the predicted environmental stress-related elements, MYB Binding Site is known to mediate plant abiotic stress. For example, TaMYB4 binds to the MYB Binding Site in the *TaPHT1;9, TaPHT1;3, TaPHT1;6* and *TaPT2* genes promoter, thus activating *TaPHT1;9, TaPHT1;3, TaPHT1;6* and *TaPT2* genes transcription and positively regulating phosphate responses (Wang et al., 2021). This may also imply that TaMYBs may also be involved in the regulation of *TaMRS2/CorA/NIPA*-mediated magnesium ion transport in wheat.

### Protein–protein interaction network of magnesium transporter genes

**Figure 7:**
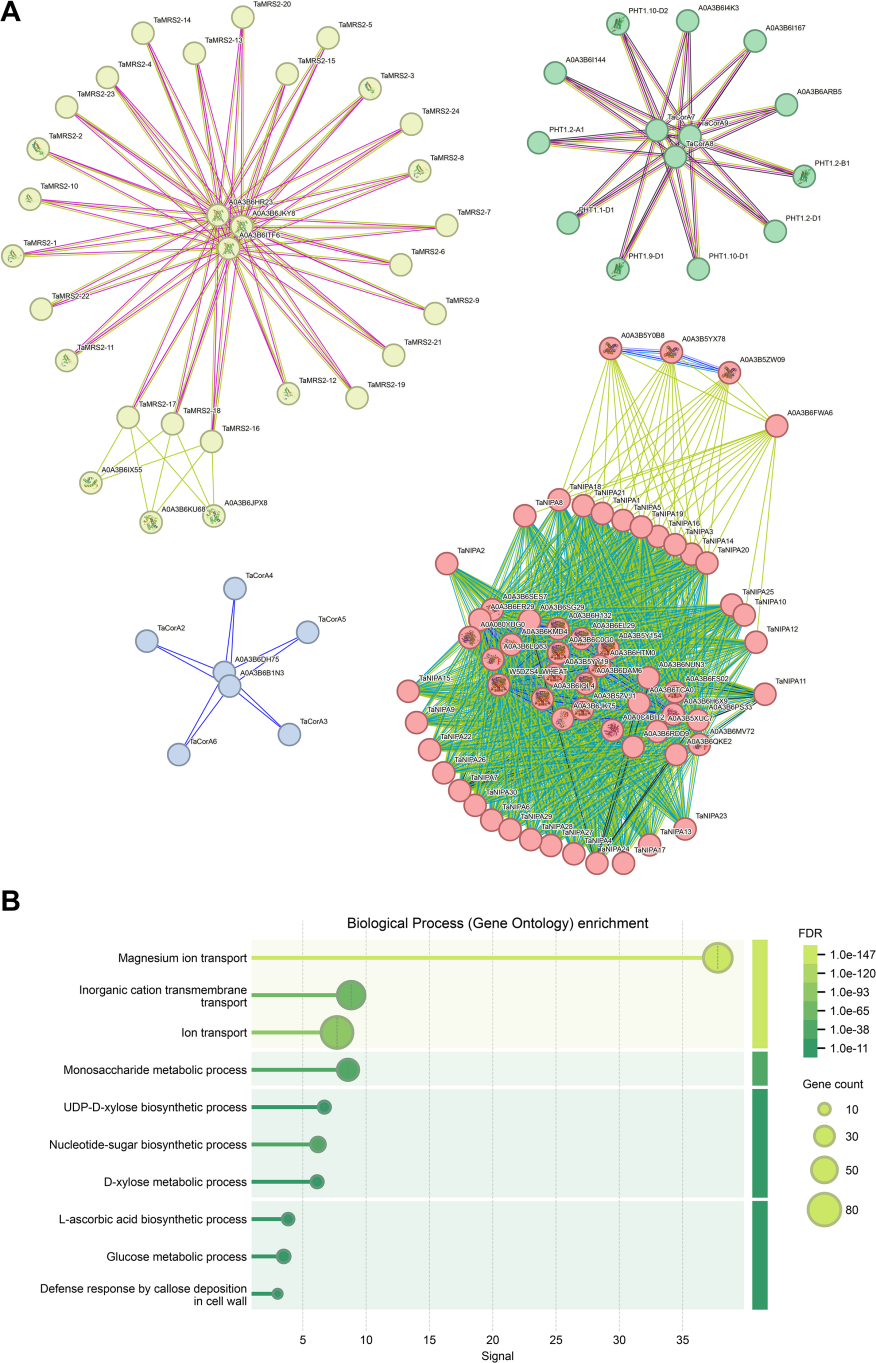
Protein association analyses of TaMGT proteins. **(A)** Predicted protein association networks analyses of TaMGT proteins. The four colors represent different interaction areas. The nodes represent the proteins, and the lines represent the protein–protein associations. Light blue and purple lines represent the known interactions from the curated database or experimentally determined interactions; green, red, and blue lines represent gene neighborhood, gene fusions, and gene co-occurrence, indicating that the proteins have the predicted interactions; yellow, black, and light blue lines represent textiming, co-expression, and protein homology, respectively. **(B)** Biological process (gene ontology) enrichment analysis of proteins in the interacting network.

To understand protein–protein interactions between magnesium transporter proteins and other proteins in wheat, we constructed a protein–protein interaction network (**Figure 7**; **Supplementary Table 3**). The results showed that a total of 62 TaMRS2/CorA/NIPA proteins and 1045 interacting protein branches were identified. All TaMRS2 proteins may be interact with A0A3B6HR23, A0A3B6ITF6, and A0A3B6JKY8 proteins. Subsequent analysis showed that all three proteins possessed three Mito_carr domains (**Figure 7**; **Supplementary Table 4**). This suggesting that these proteins play a significant role in the regulation of TaMRS2 protein networks. Some TaCorAs, such as TaCorA7, TaCorA8, and TaCorA9 could interact with up to many PHT1 proteins. Other TaCorA proteins may interact with A0A3B6DH75 and A0A3B6B1N3 proteins. TaNIPA proteins has the most interacting proteins that we predicted. By analyzing the domains of interacting proteins, it is found that most of them have PLN domains. This suggesting that PLN domains may play a significant role in the regulation of TaNIPA protein networks.

To further confirm the function of interacting proteins, we performed biological process (gene ontology) enrichment analysis of proteins in the interacting network. The results showed that interacting network proteins were mainly enriched in monosaccharide metabolic process, UDP-D-xylose biosynthetic process, and nucleotide-sugar biosynthetic process. This also indicates that magnesium ion transporters in wheat mainly play an important role in the pathway of glucose metabolism (**Figure 7**).

### Expression patterns of magnesium transporter genes during growth and development

**Figure 8:**
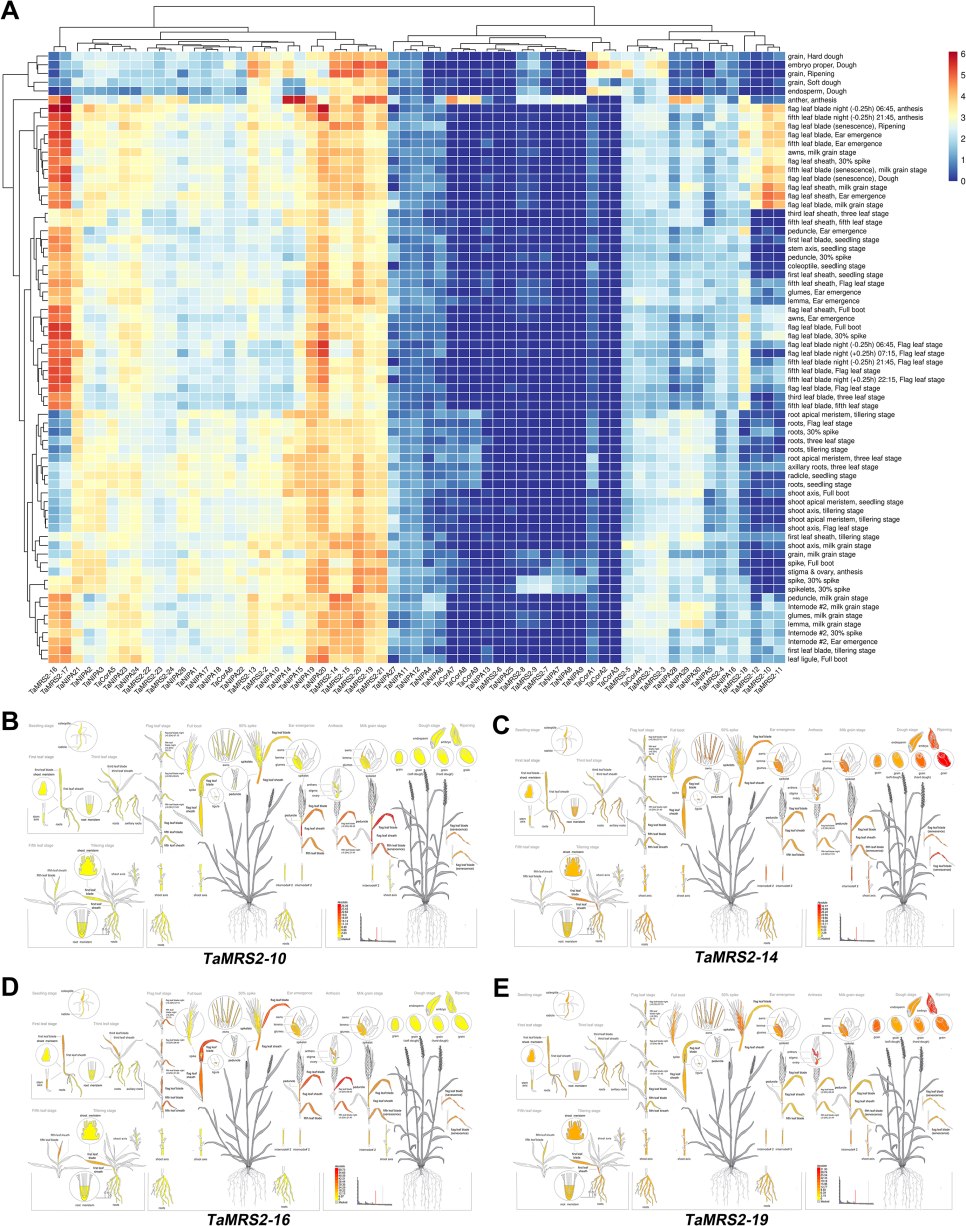
Transcriptome analyses of *TaMGTs* in different tissues. **(A)** Heat map of expression profiles for 63 *TaMGT* genes in different tissues. Red color indicates high expression levels; blue color indicates low expression levels. The gradual change of the color indicates different levels of gene log2-transformed expression. **(B-E)** Using BAR online expression analysis, create an “electronic fluorescence pictogram” representation of the target *TaMRS2-10* **(B)**, *TaMRS2-14* **(C)**, *TaMRS2-16* **(D)**and *TaMRS2-19* **(E)** genes expression pattern based on the Wheat Atlas dataset.

To generate expression profiles of the magnesium transporter genes under normal conditions, RNA sequence transcriptome data were collected and analyzed. Systematic clustering analysis was performed based on the log^2^ of TPM values for 63 magnesium transporter genes (**Figure 8**; **Supplementary Table 6**). The data showed that magnesium transporter gene expression showed great differences with the change in the growth period. The highest expression level was found in the anther, a total of 82.54% (52/63) the *TaMGT* genes were highly expressed (TPM values >1). The lowest expression level was found in the endosperm of the dough stage, a total of 50.79% (32/63) the *TaMGT* genes were highly expressed. Most of the *TaMGT* genes were expressed in all tissues, although *TaNIPA13 TaMRS2–6* and *TaNIPA2* were only expressed at low levels. Additionally, *TaMRS2–16* and *TaMRS2–17* were continuously expressed at high levels in leaf anther and spike. *TaNIPA19, TaNIPA20, TaMRS2-14, TaMRS2-15, TaMRS2-19, TaMRS2–20* and *TaMRS2–21* were continuously expressed at high levels in all tissues, suggesting that these genes may be important for plant growth and organ development (**Figure 8A**). *TaMRS2-12, TaMRS2–10* and *TaMRS2–11* were continuously expressed at high levels in flag leaf blade in different stage, suggesting that these genes may play an important role in magnesium ion transport in flag leaves of wheat. Interestingly, in order to better understand the magnesium transporter genes expression during growth and development, BAR online software was used to display the electron fluorescence diagram of *TaMRS2-10, TaMRS2-14, TaMRS2–16* and *TaMRS2–19* expression. Our results suggest that some *TaMRS2s* may play important role in many biological processes during wheat growth.

**Figure 9:**
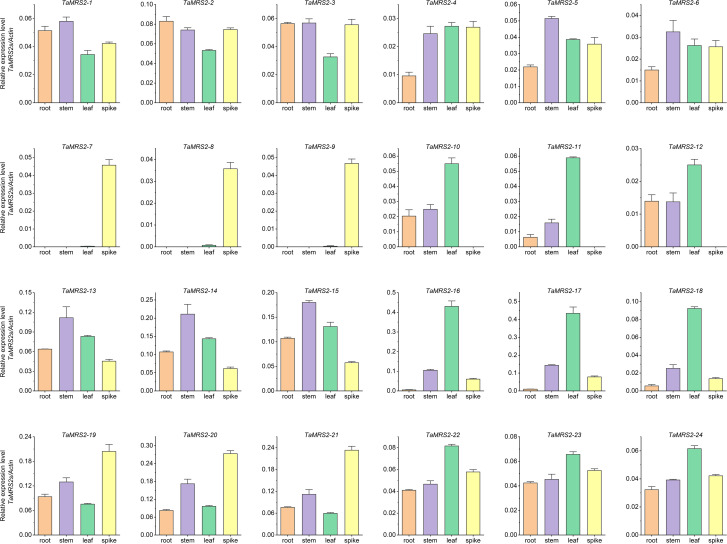
Relative expression levels of 24 *TaMRS2* genes in different tissues. Ordinate coordinates are assigned using *TaActin* as a reference.

To further investigate the possible functions of *TaMRS2s*, qRT-PCR was used to measure the expression patterns of 24 *TaMRS2* genes in four tissues (roots, leaves, stems, and spikes) (**Figure 9**). Twenty-four *TaMRS2s* were expressed in the four tissues, with strong tissue-specific expression patterns. The number of TaMRS2 genes that were highly expressed in leaves, stems and spikes was higher than that in roots. For example, 9 genes (*TaMRS2-10, -11, -12, -16, -17, -18, -22, -23* and *-24*) showed high expression levels in leaves, 6 genes (*TaMRS2-7, -8, -9, -19, -20* and *-21*) were expressed in spikes, and 5 genes (*TaMRS2-5, -6, -13, -14* and *-15*) were mainly found in stems, and no genes showed high expressions in roots. This suggests that the *TaMRS2* gene plays a major role in the leaves, stems and roots, and different *TaMRS2* genes play specific roles in different tissues of wheat.

### SNP analysis of TaMRS2-13, TaMRS2-14, TaMRS2-19, and TaMRS2-20

Single Nucleotide Polymorphism (SNP) mainly refers to the DNA sequence polymorphism caused by the variation of a single nucleotide at the genome level. It is one of the most common types of genetic variation in living organisms. Mutations in certain SNPs can cause differences in function and even traits.

**Figure 10:**
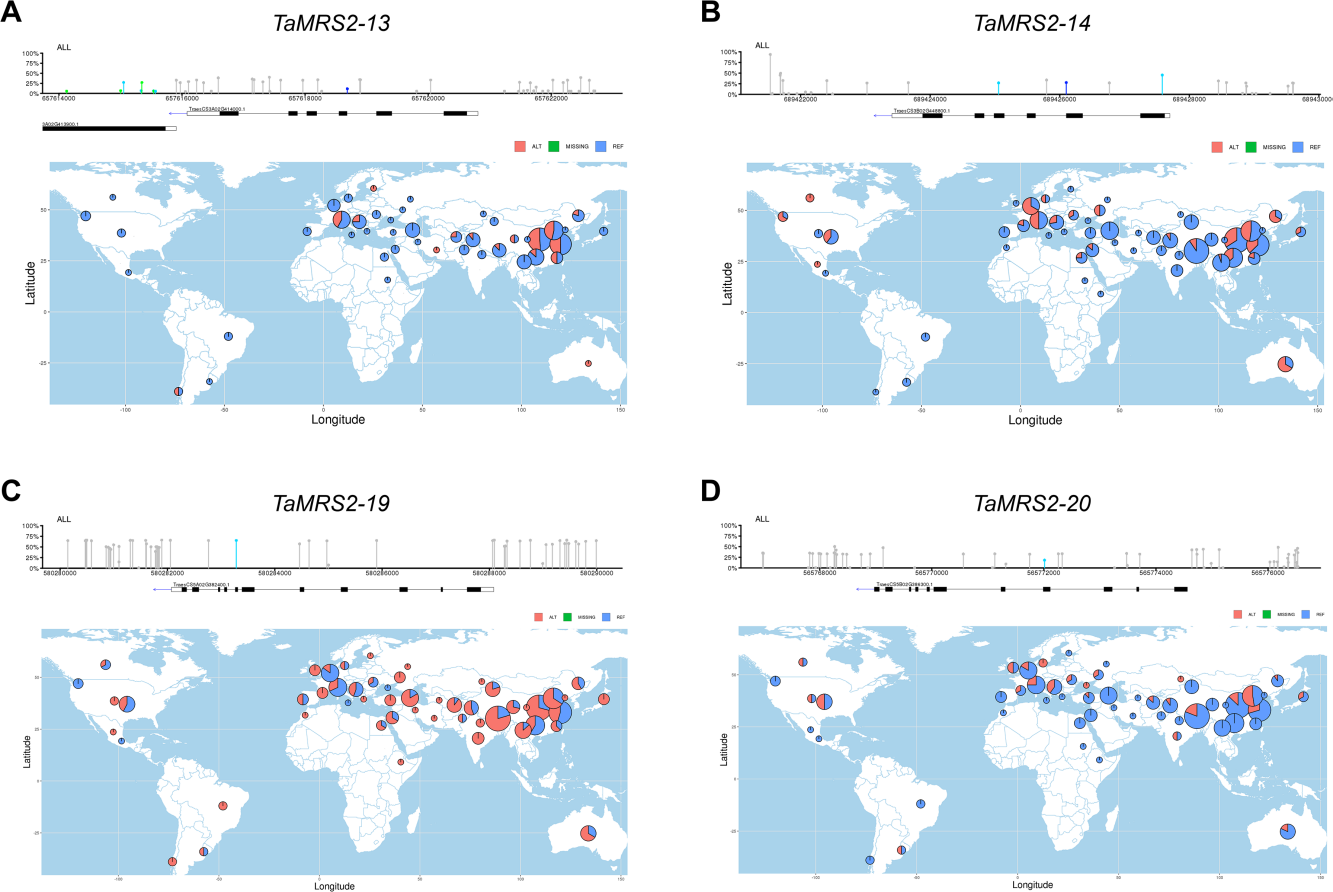
**(A-D)** SNP analysis and global distribution analysis of major variant loci of *TaMRS2-13, TaMRS2-14, TaMRS2-19, and TaMRS2-20.* **(A)** SNP analysis and the variation of chr3A:657616238 in *TaMRS2-13;* **(B)** SNP analysis and the variation of chr3B:689425056 in *TaMRS2-14*; **(C)** SNP analysis and the variation of chr5A:580280143 in *TaMRS2-19;* **(D)** SNP analysis and the variation of chr5B:565772013 in *TaMRS2-20*.

This is of great significance to the study of gene function in organisms. Here we analyzed SNPs of coding region and 3000bp before and after the gene in *TaMRS2-13, TaMRS2-14, TaMRS2-19*, and *TaMRS2-20*. We found SNPs in the exons of the coding regions of the four genes, among which a large number of SNPs were also present in the promoter region of *TaMRS2-13, TaMRS2-19*, and *TaMRS2-20*, while a small number of SNPs were present in the promoter region of *TaMRS2-14* (**Figure 10**).

Then we analyzed the distribution of the major SNPS in these genes in 1769 materials worldwide. The results show that the variation of chr3A:657616238 in *TaMRS2–13* was mainly distributed in China and parts of Europe, and the variation frequency was low, accounting for 20.14% of all materials. The variation of chr3B:689425056 in *TaMRS2–14* was mainly distributed in China, Japan, Australia, Europe and North America, and the variation frequency was high, accounting for 41.20% of all materials. The variation of chr5A:580280143 in *TaMRS2–19* was worldwide, and the variation frequency was very high, accounting for 73.32% of all materials. The variation of chr5B:565772013 in *TaMRS2–20* was mainly distributed in East Asia, North America and Europe, and the variation frequency was low, accounting for 23.89% of all materials.

### Subcellular localization of TaMRS2-1/2/3, TaMRS2-16/17/18, and TaMRS2-19/20/21

**Figure 11:**
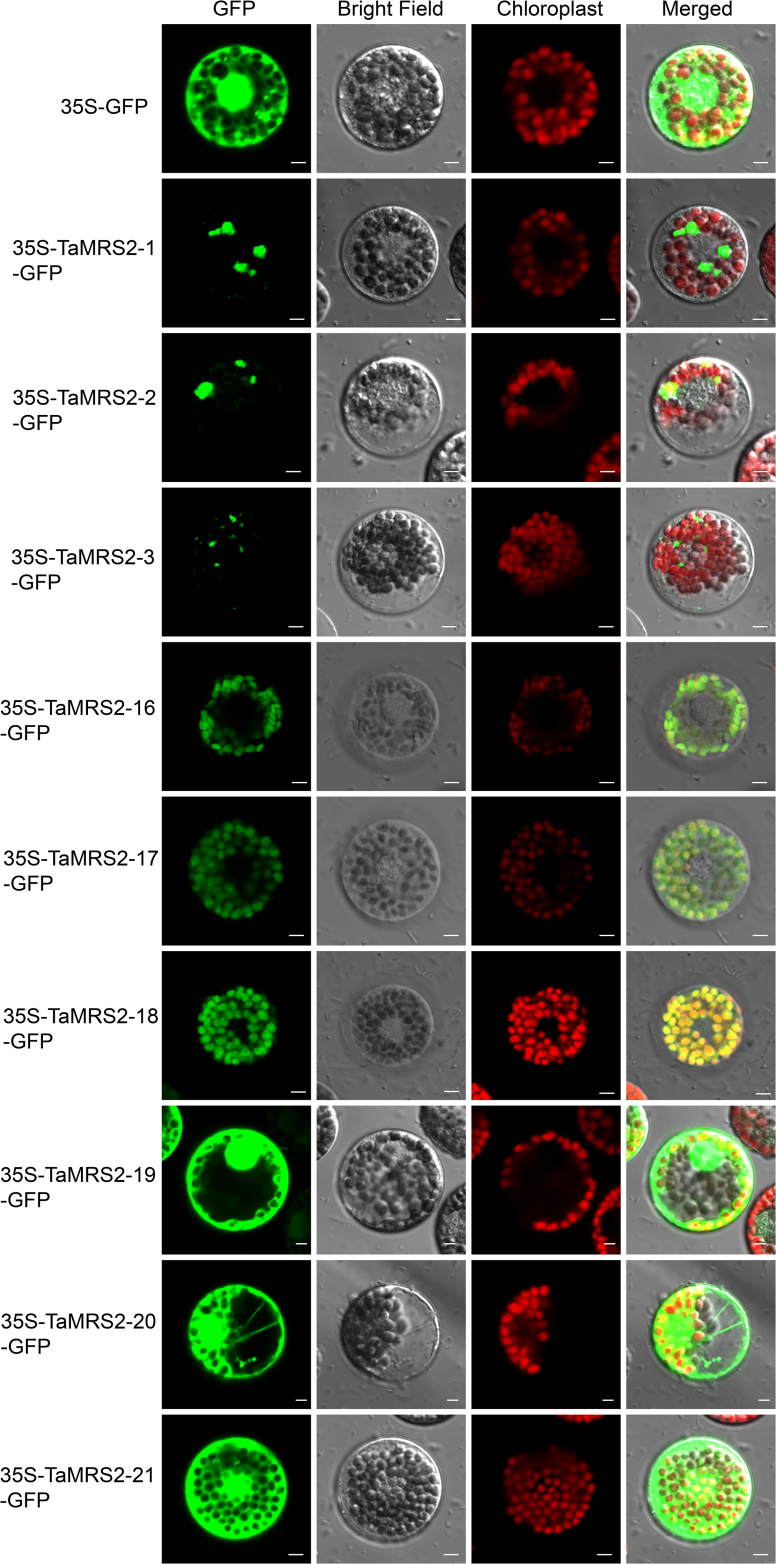
The subcellular location of TaMRS2-1, TaMRS2-2, TaMRS2-3, TaMRS2-16, TaMRS2-17, TaMRS2-18, TaMRS2-19, TaMRS2–20 and TaMRS2-21. Localization of TaMRS2 proteins under normal conditions. Images were observed under a confocal laser scanning microscope (LSM 700, Zeiss). Scale bars = 10 μm.

To better understand the functions of *TaMRS2s*, nine *TaMRS2* genes with high expression levels and different expression patterns were selected for subcellular localization analysis (**Figure 11**). The coding sequence of the *TaMRS2-1/2/3, TaMRS2-16/17/18*, and *TaMRS2-19/20/21* genes were fused with the N- or C-terminus of GFP, driven by the Cauliflower mosaic virus (CaMV) 35S promoter. The results showed that the subcellular localization of different types of *TaMRS2* genes was different, TaMRS2-16/17/18 were located in the chloroplast, TaMRS2-19/20/21 were located in the whole cell, and had strong GFP fluorescence signal. The subcellular localization of TaMRS2-1/2/3 was found in irregular organelles, which needs to be further explored. Although we predicted the subcellular localization of these genes, such as the predominant distribution in chloroplast and organelle membranes. However, the subcellular localization of different types of TaMRS2 is different in wheat.

## Discussion

### Characteristics of magnesium transporter family genes in wheat

Magnesium transporters are key factors in maintaining ion balance and play an important role in regulating plant growth and development (Tian et al., 2021; Ishijima, 2021). Since the first Mg^2+^ transporter gene, *AtMHX1* (AT2G47600), was identified in *Arabidopsis*, Mg^2+^ transporter family genes characterized in an increasing number of plant species, including rice, maize, pear, sugarcane, citrus, *brassica rape*, *camelina sativa*, grape and apple (Saito et al., 2013; Li et al., 2016; Zhao et al., 2018; Wang et al., 2019; Liu et al., 2019; Zhang et al., 2019; Faraji et al., 2021; Ge et al., 2022; Bao et al., 2025). Due to the lack of systematic analysis, the exact number of Mg^2+^ transporter family genes in the wheat genome remains unclear. Here, we systematically characterized the gene structure of the Mg^2+^ transporter family in wheat. In this study, a total of 63 Mg^2+^ transporter family gene members were identified in the wheat genome (**Table 1**). Phylogenetic analysis revealed that the Mg^2+^ transporter gene family can be divided into three large subfamilies, including NIPA, CorA and MRS2. Consistent with previous studies on magnesium ion transporters, this result further confirms the conserved divergence of these three major subfamilies within the plant magnesium ion transporter family (Regon et al., 2019; Heidari et al., 2022). Meanwhile, the molecular weights of the proteins encoded by each subfamily of wheat Mg²^+^ transporter genes show significant differences. Among them, the CorA subfamily has a relatively large molecular weight ranging from 51.78 to 63.84 kDa, the MRS2 subfamily has a medium molecular weight ranging from 39.69 to 51.16 kDa, and the NIPA subfamily has a relatively small molecular weight ranging from 33.36 to 45.49 kDa. At the same time, their differences are also reflected in hydrophobicity, most TaMRS2 proteins are hydrophilic proteins, while TaNIPA proteins are hydrophobic proteins. These differences are also reflected in magnesium ion transporters in species such as *Citrullus lanatus* and *Cucumis sativus* (Heidari et al., 2022). It is precisely these differences in protein properties that lead to the differentiation of different subfamilies in their subcellular localization and functions within the cell membrane.

In terms of subcellular localization, the gene products of wheat magnesium ion transporters were mainly predicted to localize to sites such as the cell membrane and vacuolar membrane. This is basically consistent with their localization in species like *Arabidopsis thaliana* and rice (Saito et al., 2013; Li et al., 2016), indicating that the function of magnesium ion transporters in the intracellular membrane system is conserved across different species—all of them are involved in the regulation of intracellular magnesium ion homeostasis and related physiological processes. Notably, however, a small number of magnesium ion transporters in wheat (e.g., TaMRS2-16/17/18) are localized near the chloroplast membrane. This shares a similar localization with the important magnesium ion transporter OsMGT3/OsMRS2–6 in rice (Li et al., 2020). Therefore their potential functions near the chloroplast membrane, such as the precise regulation of magnesium ion supply during photosynthesis, deserve in-depth investigation.

### Evolution of magnesium transporter family genes in wheat

The number of Mg^2+^ transporter family gene members in wheat appeared to be threefold higher than the 22 family members in diploid *Arabidopsis*. This may be attributed to the fact that wheat is an allohexaploid, whose origin involved two polyploidization events, resulting in a threefold increase in gene number compared to diploid species (Wang et al., 2025). However, when subdivided into each subfamily, the MRS2/MGT subfamily expanded by less than threefold, while the NIPA subfamily expanded by fourfold or more. This also suggests that the divergence of the Mg^2+^ transporter gene family in wheat species may contribute to the specificity of Mg^2+^ transport in this species. Phylogenetic trees were also constructed for 116 *MRS2* genes, including five *MRS2* genes from monocot species and three from dicot species. The phylogenetic tree showed that the *MRS2* genes were distributed across five distinct clades (designated as branches A-E in the figure). The results indicated that *MRS2* genes from each clade were present in all eight species analyzed, demonstrating that *MRS2* genes in each branch are highly conserved among plants. Meanwhile, branch B *MRS2* genes were significantly expanded in monocot plants; especially in wheat, there were 9 branch B *MRS2* genes, accounting for 37.5% of the total *TaMRS2* genes, whereas branch C *MRS2* genes were significantly expanded in dicot plants. This also indicates divergent differences in *MRS2* genes between monocots and dicots. This also indicates divergent differences in *MRS2* genes between monocots and dicots, which may contribute to the differences in Mg²^+^ transport between these two major plant groups. We also analyzed the chromosomal distribution of the Mg^2+^ transporter gene family in wheat. Interestingly, all nine TaMRS2 genes from clade B were located on chromosome 3, where they formed a gene cluster consisting of three *TaMRS2* genes, two *TaCorA* genes, and six members of the NIPA subfamily. Whether this gene cluster plays an important role in Mg^2+^ transport in wheat requires further molecular investigation.

Gene duplication events are crucial for the rapid expansion and evolution of plant gene families (Walkowiak et al., 2020). Studies have shown that more than 85% of the genome sequence of common wheat (*Triticum aestivum L.*) consists of repetitive sequences (Fernández Gómez and Wilson, 2014). Collinearity analysis (**Figure 4**) revealed a large number of segmental duplication events during the evolution of *TaMGTs* genes, indicating that segmental duplication contributes to the amplification of *TaMGT* gene family. Concurrent with this, during the evolutionary process, wheat magnesium ion transporter genes exhibit both gene sharing and gene differentiation with closely related species. Certain ancient magnesium ion transporter genes are highly conserved between wheat and its relatives, for example, *TaMRS2-4/5/6, TaMRS2-13/14/15* and *TaCoA4/5/6* exhibit extremely high colinearity in barley, rice, and maize. This may be because these genes perform essential and conserved functions in fundamental plant physiological processes, such as maintaining normal cellular osmotic pressure and participating in numerous enzymatic reactions, thereby being subjected to strong selective pressure (Wang et al., 2021). Conversely, certain wheat-specific magnesium transporters, such as *TaMRS2-16/17/18, TaMRS2-19/20/21*, and a substantial proportion of *TaNIPA* genes, have undergone rapid evolution. Significant changes in the sequence and structure of these genes may be associated with wheat’s adaptation to specific environments or the acquisition of new biological functions (Liu et al., 2021). Collinearity and interspecific evolutionary relationship analysis of wheat *TaMGTs* genes among species (**Figure 4**) showed that among monocot species rice (13.67Mya), barley (16.00Mya) and maize (51.56Mya) diverged sequentially, indicating that wheat has a more similar gene structure to rice.

### Functional analysis and regulatory significance of the protein-protein interaction network

Protein synergistic interaction is the core molecular basis of Mg^2+^ transport regulation. The TaMRS2/CorA/NIPA protein interaction network constructed in this study (**Figure 7**; **Supplementary Table 3**) provides key clues for analyzing the regulatory mechanism. The network identified 62 target transporters and 1045 interaction branches, among which all TaMRS2 proteins interact with A0A3B6HR23, A0A3B6ITF6, and A0A3B6JKY8 proteins containing three Mito_carr domains. Mito_carr domains are mostly found in mitochondrial carrier proteins (Tang et al., 2015). Combined with the subcellular localization results, it is speculated that these domains mediate the functional association between TaMRS2 and mitochondria, participate in the regulation of mitochondrial Mg homeostasis, thereby affect ATP synthesis and photosynthetic product accumulation.

Notably, transporters from different subfamilies exhibit distinct interaction characteristics, revealing the specificity of their functional division. For instance, members such as TaCorA7/8/9 interact with multiple key phosphorus transport proteins PHT1. Since there are synergistic or antagonistic effects between Mg and phosphorus absorption and utilization (Hui et al., 2024). It is suggested that their interaction may mediate the regulation of wheat Mg-phosphorus nutritional balance, providing new targets for the analysis of mineral nutrient synergistic mechanisms. The number of interactions of TaNIPA proteins exceeds expectations, and most interacting proteins contain PLN domains related to ion transport, indicating that PLN domains are key modules for TaNIPA-mediated Mg^2+^ transport regulation. Their interaction networks may be involved in the adaptive regulation of wheat to Mg nutritional stress.

GO enrichment analysis showed that the proteins in the interaction network are mainly enriched in monosaccharide metabolism, UDP-D-xylose biosynthesis, and nucleotide-sugar biosynthesis processes (**Figure 7**), confirming that wheat Mg^2+^ transporters not only regulate Mg^2+^ homeostasis but also may involved in glucose metabolism pathways. Therefore, we speculate that magnesium transporters can regulate enzyme activity or substrate supply through interaction with glycolysis-related proteins, thereby affecting the synthesis and distribution of photosynthetic products.

### Expression patterns and potential function of the magnesium transporter gene family in wheat

Genes exert their functions through transcription and translation, with their expression patterns reflecting their functional roles. Previous studies have demonstrated that the expression patterns of magnesium ion transporter gene families vary among different species and are concentrated in distinct tissue types (Zhang et al., 2019; Faraji et al., 2021; Bao et al., 2025). For instance, in sugarcane (*Saccharum*), *SsMTG9* is one of the most highly expressed Mg transporters in mature leaf regions, playing a crucial role in functional photosynthetic areas, while *SsMGT3* participates in magnesium homeostasis within mature stems and leaves during photoperiods (Wang et al., 2019). In sorghum, *SbMGT4* and *SbMGT5* exhibit elevated expression levels in reproductive organs, specifically the pistil and anther (Davidson et al., 2012). In *Arabidopsis*, *AtMGT6* is sensitive to magnesium deficiency, as its expression is strongly induced by low magnesium levels in cortical and epidermal cells, including root hairs (Mao et al., 2014). Additionally, both *AtMGT5* and *AtMGT9* in *Arabidopsis* are expressed in pollen tissues, where they play vital roles in Mg supply for pollen mitosis and pollen intine formation (Drummond et al., 2006; Chen et al., 2009; Li et al., 2015). In this study, we investigated the expression patterns of 62 magnesium ion transporter genes. Results revealed significant variations in *TaMGT* expression across different wheat tissues throughout the growth cycle. Notably, during the flowering stage, 46.8% of magnesium ion transporter genes exhibited high expression in anthers, suggesting these transporters may play a primary role in wheat anther function. Moreover, *TaMRS2-16/17/18* exhibits high homology with *AtMGT10/AtMRS2–11* and *OsMGT3/OsMRS2-6*, maintaining elevated expression levels throughout the entire growth cycle. In *Arabidopsis*, AtMGT10 is localized within the envelope membrane of leaf chloroplasts and is associated with Mg homeostasis within the chloroplast (Drummond et al., 2006). In rice, OsMGT3/OsMRS2–6 similarly localizes to the chloroplast envelope membrane, regulating Mg fluctuations within rice chloroplasts and the activity of ribulose-1,5-bisphosphate carboxylase in rice plants, thereby influencing photosynthetic efficiency and growth performance (Li et al., 2020). Our study reveals that TaMRS2-16/17/18 localizes to the chloroplast membrane and is highly expressed in green organs. Consequently, TaMRS2-16/17/18 may represent a potential target gene in wheat that influences chloroplast magnesium ion concentration regulation and thereby affects photosynthetic efficiency. Our study further identified three genes—*TaMRS2-7/8/9*—expressed exclusively in the spikelet region. Their highly homologous counterparts AtMGT4/AtMRS2–3 are localized to the endoplasmic reticulum and highly expressed in pollen, the *atmgt4–1* mutant exhibits a pollen abortive phenotype (Li et al., 2015). We therefore hypothesize that *TaMRS2-7/8/9* are crucial for wheat anther development. However, their developmental functions require further validation.

### Genetic significance and breeding value of SNP analysis in *TaMRS2* genes

SNP, as a key genetic variation marker, is a core tool for gene function analysis and excellent allele mining (Henry and Edwards, 2009). In this study, SNP sites were detected in the exons of the coding region and promoter region of *TaMRS2-13/14/19/20* genes (**Figure 10**). The distribution of SNPs in the promoter region showed gene specificity: *TaMRS2-13/19/20* had abundant SNPs in the promoter region, while *TaMRS2–14* had fewer. SNPs in the promoter region may regulate gene expression patterns by affecting transcription factor binding sites, and SNPs in the coding region may change amino acid sequences to affect the structure and function of transporters (e.g., Mg binding affinity, transport efficiency), indicating that the *TaMRS2* gene family has rich genetic diversity, providing core targets for mining excellent alleles related to efficient Mg utilization. Analysis of SNP distribution in 1769 global wheat accessions reveals *TaMRS2s*’ breeding potential, and the variation distribution is related to geographical environmental selection pressure: the chr5A:580280143 variation in *TaMRS2–19* is distributed globally with an extremely high frequency (73.32%), which is speculated to be a conserved excellent variation, ensuring the basic Mg transport function and wide adaptability of wheat; the chr3B:689425056 variation in *TaMRS2–14* has a high frequency (41.20%) in multiple major producing areas, which may be a key site for efficient Mg utilization under soil conditions in these areas; the specific variation frequency of *TaMRS2-13/20* is low, which may be related to local environmental adaptability. The above SNP sites provide theoretical support and technical targets for wheat Mg-efficient molecular breeding.

## Conclusions

In summary, through multi-dimensional systematic analysis, this study revealed the role of the wheat magnesium transporter family in growth and development, namely the core regulatory logic that evolution lays the foundation for function, localization and interaction determine the functional pathway, expression adapts to physiological needs, and variation is associated with breeding value. Evolutionarily, the family has achieved a threefold expansion due to the allohexaploid characteristics of wheat. Differences in subfamily expansion and clade differentiation between monocots and dicots not only retain conserved genes such as *TaMRS2-4/5/6* to ensure basic magnesium homeostasis but also evolve specific genes such as*TaMRS2-16/17/18* to adapt to specific environments, and segmental duplication enhances functional diversity. At the functional execution level, subcellular localization is closely associated with protein interaction networks: membrane-localized members ensure basic transport, *TaMRS2-16/17/18* localized in chloroplasts participate in photosynthetic regulation, *TaMRS2* is associated with energy metabolism, *TaCorA* constructs a magnesium-phosphorus synergistic pathway, and the glycolysis-related processes enriched by interacting proteins reveal the intrinsic link between magnesium transport and photosynthetic product synthesis. Expression patterns precisely match functional division: genes highly expressed in anthers at the flowering stage adapt to reproductive magnesium needs, *TaMRS2-16/17/18* highly expressed in green organs echo their photosynthetic functions, and *TaMRS2-7/8/9* specifically expressed in spikelets point to another development regulation. The key variation sites identified by SNP analysis are related to geographical selection pressure, which not only confirms the adaptive evolution of functional genes but also provides precise targets for magnesium-efficient breeding. Based on this, integrating multi-dimensional information to construct a functional regulatory model can clearly clarify the entire regulatory network of magnesium transport and its core association with photosynthesis and reproduction, deepen the understanding of the wheat magnesium nutrition regulatory mechanism, and provide a precise direction for subsequent research and breeding.

## Data Availability

The datasets presented in this study can be found in online repositories. The names of the repository/repositories and accession number(s) can be found in the article/**Supplementary Material**.
