# Supplementary figures and images for: Comparative genomics revealed the gene evolution and functional divergence of TaMRS2/CorA/NIPA magnesium transporter families in wheat (Triticum aestivum L.)
Source: Front Plant Sci. 2026 Mar 9;17:1737134. doi: 10.3389/fpls.2026.1737134 (PMC13048270; doi:10.3389/fpls.2026.1737134)

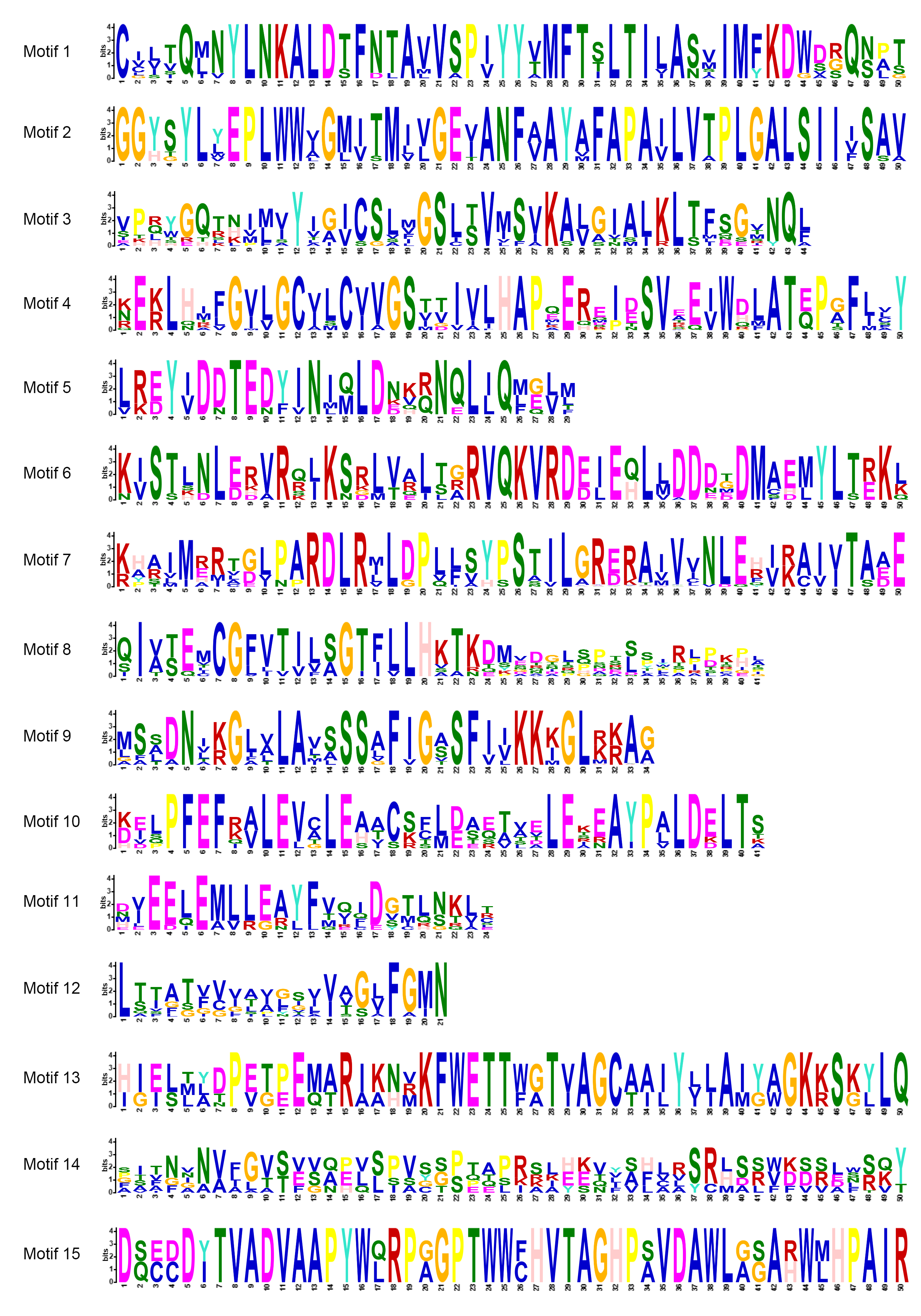

Supplement: Supplementary file 1 [file Image1.tif]
